# Population history modulates the fitness effects of Copy Number Variation in the Roma

**DOI:** 10.1007/s00439-023-02579-5

**Published:** 2023-06-14

**Authors:** Marco Antinucci, David Comas, Francesc Calafell

**Affiliations:** Institute of Evolutionary Biology (UPF-CSIC), Department of Medicine and Life Sciences, Universitat Pompeu Fabra, Barcelona, Spain

## Abstract

We provide the first whole genome Copy Number Variant (CNV) study addressing Roma, along with reference populations from South Asia, the Middle East and Europe. Using CNV calling software for short-read sequence data, we identified 3171 deletions and 489 duplications. Taking into account the known population history of the Roma, as inferred from whole genome nucleotide variation, we could discern how this history has shaped CNV variation. As expected, patterns of deletion variation, but not duplication, in the Roma followed those obtained from single nucleotide polymorphisms (SNPs). Reduced effective population size resulting in slightly relaxed natural selection may explain our observation of an increase in intronic (but not exonic) deletions within Loss of Function (LoF)-intolerant genes. Over-representation analysis for LoF-intolerant gene sets hosting intronic deletions highlights a substantial accumulation of shared biological processes in Roma, intriguingly related to signaling, nervous system and development features, which may be related to the known profile of private disease in the population. Finally, we show the link between deletions and known trait-related SNPs reported in the genome-wide association study (GWAS) catalog, which exhibited even frequency distributions among the studied populations. This suggests that, in general human populations, the strong association between deletions and SNPs associated to biomedical conditions and traits could be widespread across continental populations, reflecting a common background of potentially disease/trait-related CNVs.

**Supplementary Information:**

The online version contains supplementary material available at 10.1007/s00439-023-02579-5.

## Introduction

Structural variants (SVs) are a class of genomic rearrangements, larger than 50 bp, comprising insertions, deletions, duplications, inversions and translocations, which are responsible for the largest fraction of base pair variation in the human genome (Weischenfeldt et al. 2013; Sudmant et al. 2015b). Within SVs, balanced mutations (inversions and translocations) do not alter the genomic dosage, while unbalanced rearrangements (insertions, duplications and deletions, the latter two also known collectively as Copy Number Variants, CNVs) involve losses or gains of genetic material. CNVs can exert their influence on gene expression, phenotypic traits, and diseases, and represent a main source of genetic variation on which natural selection can act upon (Stranger et al. 2007; Hurles et al. 2008; Perry et al. 2008; Handsaker et al. 2015; Audano et al. 2019; Collins et al. 2020; Hollox et al. 2022). Indeed, CNVs have been linked to a number of traits such as Crohn’s disease, osteoporosis, HIV susceptibility, body mass index, cancers and psoriasis (McCarroll et al. 2008; Yang et al. 2008; De Cid et al. 2009; Willer et al. 2009; Mohamad Isa et al. 2020; Dentro et al. 2021; Hamdan and Ewing 2022) and are intriguingly associated to neurodevelopmental disorders in humans (Sebat et al. 2007; Stefansson et al. 2008; Girirajan et al. 2013; Singh et al. 2017; Morris-Rosendahl and Crocq 2020; Sekiguchi et al. 2020; Kato et al. 2022).

Most of the studies addressing human population genetics have historically focused on SNPs to infer human population demography, such as changes in effective population size due to bottlenecks or founder events, or gene flow due to migration. This is also the case for the investigation of the mutation load, that is, the global contribution of deleterious mutations to disease. However, research using CNVs as markers in population genetics surveys, both in large worldwide comparisons and on finer scales, has been increasingly accumulating over the last two decades and confirmed their potential in this field, highlighting among/within group variability, the functional potential of the variants (including pathogenic effects) and their evolutionary relevance (Redon et al. 2006; Itsara et al. 2008; Gautam et al. 2012; Sudmant et al. 2015b; Hehir-Kwa et al. 2016; Urnikyte et al. 2016; Dennis et al. 2017; Almarri et al. 2020; Collins et al. 2020; Bergström et al. 2020). Attention has also been given to the study of CNVs in underrepresented or isolated populations, with a putative intriguing demographic history. Earlier reports mainly focused on isolates of European ancestry, such as Scottish islands of Orkney, the Italian South Tyrol region, the Croatian Vis island and the Finnish populations. These studies highlighted the general common sharing of CNVs among population as well as isolate-specific relatedness and the presence of novel variants, uncovering a previously hidden layer of CNV variation (Chen et al. 2011; Kanduri et al. 2013). Further analyses focusing on Asian and North African samples showed enrichment of structural variant events in biomedically relevant genes (e.g., drug and wound response) (Lou et al. 2015; Romdhane et al. 2021).

The Romani or Roma population (often referred to by the problematic misnomer *Gypsies*) nowadays forms the largest transnational minority ethnic group in Europe, numbering 10–15 million. their origin has been traced back to North-western India thanks to different sources of information. Linguistic studies and records from the populations that encountered the proto-Roma groups often suggest an Indian origin of this group, which left around 1000–1500 years ago and subsequently spread to Persia and Armenia (Boerger 1984; Fraser 1992; Liégeois 1994). Records from Greece, present-day Romania, and the Czech Republic account for putative Roma presence in these territories through the fourteenth century, and by the fifteenth–sixteenth centuries, additional historical evidence documents Roma movements in many West European countries (Fraser 1992; Liégeois 1994). The current distribution of Roma people throughout Europe can be attributed to such early fifteenth-century expansions from the Balkans and to later nineteenth-century dispersals (Fraser 1992; Liégeois 1994; Reyniers 1995; Gresham et al. 2001). In more recent historical times, the Roma population size and distribution in Europe is also the consequence of the genocide they suffered, carried out by the Nazi Germany regime (Milton 1991; Lutz 1995; Sridhar 2006). Finally, the fall of the communist regimes in Central and Eastern Europe facilitated westward economically driven migrations.

The European Roma groups, indeed, have had a complex history, both in terms of the movements and contacts with different populations. Population genetics studies traced back their South Asian-related ancestry, with subsequent European admixture, from autosomal and uniparental markers (Gresham et al. 2001; Moorjani et al. 2013; Font-Porterias et al. 2019; Ena et al. 2022). Their specific history also shaped the landscape of genetic diseases, as different deleterious mutations were detected at higher frequencies, while other mutations are absent or at lower frequencies compared to other non-Roma populations (Kalaydjieva et al. 2001; Morar et al. 2004; Mendizabal et al. 2013). Specifically, private disease-causing mutations, highlighting a scenario typically found in a founder population, have been identified also in the Roma. The traits associated to these mutations are, among others, polycystic kidney disease, congenital glaucoma, congenital myasthenia, galactokinase deficiency, different neuropathies and centronuclear myopathy (Kalaydjieva et al. 1996, 1999, 2001; Piccolo et al. 1996; Angelicheva et al. 1999; Morar et al. 2004; Cabrera-Serrano et al. 2018).

The whole genome sequence of 46 Roma individuals revealed a strong, early founder effect followed by a drastic reduction of ∼44% in effective population size (*N*_*e*_) (Bianco et al. 2020). It is known that mutations reach fixation faster in small populations due to drift and, as a consequence, some deleterious mutations may rise in frequency and, under specific conditions (see Fig. 1 in Kimura et al. 1963), slightly deleterious variants can result in a larger load than more deleterious ones (Kimura et al. 1963; Kimura and Ohta 1969). In general, a rule of thumb is that drift will prevent the removal of deleterious mutations if *N*_*e*_*s* < 1, where *s* is the selection coefficient; still, this does not encompass the complexities of population growth and gene flow (Gazave et al. 2014; Lohmueller 2014). Different studies not only observed these phenomena in general populations as the Europeans, but also confirmed them in smaller and isolated groups which experienced more recent bottlenecks (i.e., Finnish, French-Canadians, Inuit and Ashkenazi Jewish) (Kaklamani et al. 2008; Lohmueller et al. 2008; Thaler et al. 2009; Casals et al. 2013; Lim et al. 2014; Pedersen et al. 2017). Moreover, disease-associated variants show specific haplotype ancestry backgrounds in Roma (European or South Asian), in line with the mutual contribution of these ancestries to Roma genetic makeup and, additionally, that the higher frequencies of SNPs mapping to drug-binding domains match the population higher proportion of diseases targeted by such drugs (Font-Porterias et al. 2021). This stresses how admixture dynamics, demographic history and the functional role of variants all contribute to the shaping of the extant diversity detectable nowadays in Roma.

**Fig. 1:**
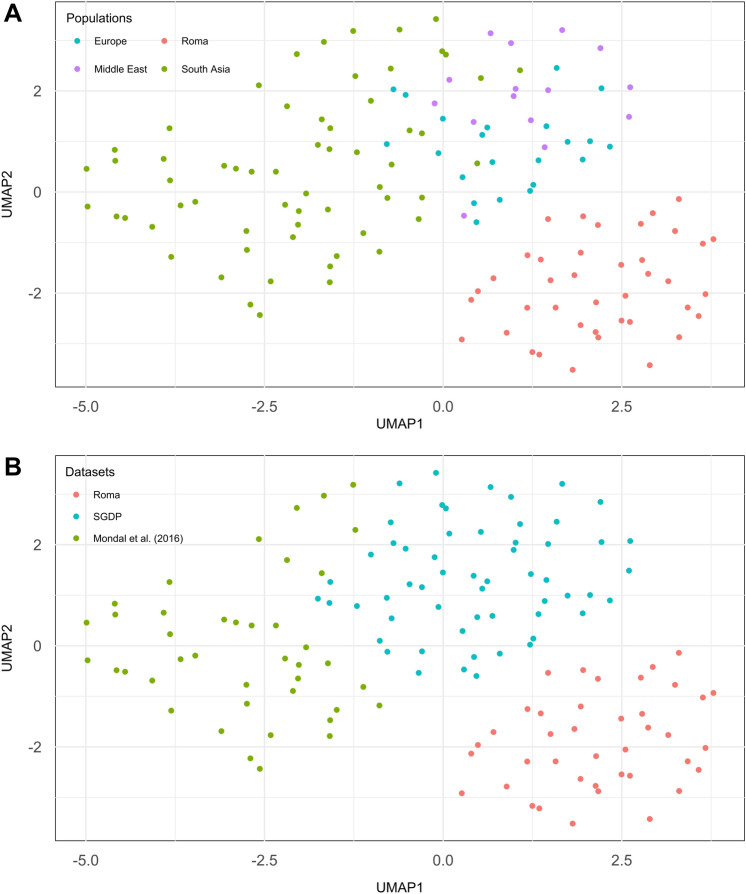
UMAP plots for deletions copy numbers. UMAP plots representing samples dataset labeled with regional assignation (**A**) and dataset of origin (**B**)

In light of the information about Roma gathered so far, we hereby analyze for the first time CNVs in high-depth complete genomes from the underrepresented European Roma population to understand how their demographic history may have contributed (if at all) to their mutation spectrum and mutational load.

## Materials and methods

### Samples

Our study comprises 40 complete genomes of Roma people collected in five European countries (Spain, Lithuania, Hungary, Ukraine and Macedonia) and belonging to four major migrant groups: 15 North/Western, 5 Vlax, 10 Romungro and 10 Balkan as defined in a previous study (Bianco et al. 2020). Donors signed an informed consent and the project was approved by the Institutional Review Board of the Comitè Ètic d’Investigació Clínica-Institut Municipal d’Assistència Sanitària (CEIC-IMAS) in Barcelona, Spain, (2016/6,723/I). All participants self-identified as Roma and appropriate consent was obtained from all donors. The study was approved by our IRB (Comitè d’Ètica de la Investigació, Parc de Salut Mar, Barcelona) on June 7th 2016 (reference 2016/6723/I) and renewed on January 15th, 2020 (reference 2019/8900/I). Preliminary results were presented to the Roma community in a meeting on February 1st 2019 in Barcelona. All methods in this study were performed following the standard guidelines and regulations. Genome sequences were those analyzed in (Bianco et al. 2020), which fastq files had been deposited at the European Genome Archive with accession number EGAS00001004287. Reference samples with geographic origins matching the Roma diaspora comprised two main datasets: the Simons Genome Diversity Project (SGDP; samples from Europe, the Middle East and Pakistan) (Mallick et al. 2016) and Mondal et al. (Mondal et al. 2016) (samples from India) (see supplementary Table 5). Throughout this manuscript, when we use term *European* to refer to reference samples, we mean it as shorthand for *non-Roma Europeans*, and we do not imply that Roma should not be regarded as Europeans.

### Structural variant calling

We selected a set of six different programs using algorithms based on different strategies to detect SVs from short read sequencing data, combining the strengths of each algorithm and integrating them. Our set is composed of CNVnator (version 0.4.1) (Abyzov et al. 2011), BreakDancer (version 1.4.5) (Chen et al. 2009), Pindel (version 0.2.5b8) (Ye et al. 2009), Tardis (version 1.0.4) (Soylev et al. 2017), Lumpy (version 0.2.13) (Layer et al. 2014), and GenomeSTRiP (version 2.00.1918) (Handsaker et al. 2011, 2015) callers, which implement read-depth, split-read and read-pair methods. See Supplementary Methods for the implementation of each method.

### Data merging

We designed custom scripts to obtain the data both for the results for all callers for a single sample and among all samples. To do so, we first merged the output of the different software for each sample, specifically by merging those SVs residing on the same chromosome, deletions and duplications separately, with a reciprocal genomic coordinate overlap of at least 50% of their length. By doing so, we created clusters of overlapping pairs of calls and for each cluster (ranging from a pair of calls for two programs, up to the 15 possible pairs among six different callers) we selected the coordinates and the genotype of the most confident caller, based on the evaluation of caller performance in (Kosugi et al. 2019). Using this information for each cluster of calls mentioned above, we obtained a single call by retaining the best performing software for coordinates and genotype, respectively. To merge variants across samples, we proceeded in a similar manner as previously presented, where we joined all sample calls if variants of the same type resided on the same chromosome and reciprocally overlapped at least for 50% of their length. This allowed us to create a consensus set of calls listing the sharing of each variant among individuals.

### Additional filters

We regenotyped the CNVs of each sample with a dedicated software, GraphTyper2 (version 2.5.1) (Eggertsson et al. 2019), to accurately recover more reliable genotypic information (see supplementary methods). We further filtered the results according to the best practices as described by the software authors, to retain only good quality genotypes. To additionally filter for false positives, we used the HardyWeinberg R package (version 1.7.2) (Graffelman 2015) to remove variants violating Hardy–Weinberg equilibrium. We computed the chi-squared test *p*-value for each CNV in each population and filtered out variants having a significant result after Bonferroni correction for multiple tests. Finally, we implemented an R package algorithm leveraging SNP data to infer reliable CNVs: CNVfilteR (version 1.8.0), which detects false positive heterozygous deletions and duplications by evaluating the frequencies of SNPs mapping to each variant (Moreno-Cabrera et al. 2021). We ran this software with default parameters and obtained a set of variants indicating false positive results that were subsequently filtered out from the dataset.

### Statistical analysis

Principal component analysis was carried out using the smartpca algorithm within the Eigensoft package (version 6.0.1) (Patterson et al. 2006). Briefly, based on CNV genotypic calls, we coded biallelic deletions and duplications as zero, one, and two copy numbers and used those as input for the software to perform PCA on our samples. We additionally used another dimensionality reduction method, the uniform manifold approximation projection (UMAP) (McInnes et al. 2018) on copy number for deletions and duplications. Population structure was further assessed using ADMIXTURE (version 1.3) (Alexander et al. 2009), running 10 random seeds for each ancestral component (*K*: 2–10), to evaluate ancestry profiles among the studied samples. We filtered out variants with minor allele frequency < 0.01 and violating structure-aware Hardy–Weinberg equilibrium before running the analysis, as best practices described in previous studies (Narang et al. 2014; Hao and Storey 2019; Linck and Battey 2019). Pong (Behr et al. 2016) was used to visualize ADMIXTURE results by representing Q matrices for modes in each value of K. ANOVA test was performed with the R *car* package (version 3.0.10) (Fox and Weisberg 2011), while Kruskal–Wallis and Chi-squared tests were computed using the corresponding native R functions (R Core Team 2003). We estimated global differentiation values calculating F_ST_ statistics among pairwise populations using the StAMPP R package (Pembleton et al. 2013) and estimated p-values by performing 10,000 bootstraps. Taking advantage of the possibility to recapitulate population differentiation using CNVs data by means of the Vst statistic (Redon et al. 2006; Sudmant et al. 2015a), using a custom script, we implemented a variation of the formula described in a previous study (Serres-Armero et al. 2021), comparing directly copy number variance rather than log_2_ ratios from CGH array data. We applied the statistic in pairwise population comparisons computing the differentiation for each CNV individually.

### Copy Number Variant annotation

We used the software AnnotSV (version 3.0.7) (Geoffroy et al. 2018, 2021) for multiple database annotation to retrieve the possible clinical or functional roles of the CNVs in our dataset. Since results from AnnotSV provided different information, we focused on: (1) the genes intersected by the CNV, (2) whether the intersection involved an intron, an exon, or both, (3) diseases associated to the intersected gene provided by OMIM catalog (Hamosh et al. 2005), (4) gene tolerance to loss of function. Specifically, the tolerance to loss of function for genes intersected by CNVs is ranked as Loss-of-function Observed/Expected Upper Fraction (LOEUF) bins (range 0–9) from genomAD database (Karczewski et al. 2020). The LOEUF metric refines over the widely used pLI (probability of Loss of function Intolerance), providing a continuous rather than a dichotomous scale (e.g., pLI < 0.9; pLI > 0.9). We carried out permutation tests to screen for possible intra-population higher/lower than expected abundance of deletions intersecting intronic portions of loss of function (LoF) intolerant genes. To do so, we downloaded the LOEUF information for each gene present in the gnomAD database and obtained those genes’ annotations via Ensembl database (version 86) (Cunningham et al. 2022) using the EnsDb.Hsapiens.v86 and ensembldb R packages (Rainer 2017; Rainer et al. 2019). For this list of genes we extracted the intronic coordinates using GenomicFeatures R package (Lawrence et al. 2013) of those genes with a LOEUF ≤ 4 (Lof intolerant) and LOEUF > 4 or not reported (LoF tolerant). Then, with our list of population-specific gene-intersecting deletions and introns coordinates of LoF tolerant/intolerant genes, we performed permutation tests separately in each population using the regioneR R package (Gel et al. 2016) performing 5000 permutations and estimating the numOverlaps and randomizeRegions as the evaluate and randomize functions.

### Over-representation analysis

To assess putative significant enrichment in biological pathways for our gene-intersecting SVs, we interrogated the Gene Ontology Resource (Ashburner et al. 2000) using the WEB-based GEne SeT AnaLysis Toolkit (WebGestalt) (Zhang et al. 2005; Liao et al. 2019), an online tool to interpret and analyze gene lists of specific interest. We tested whether the list of genes classified with a LOEUF score from 0 to 4 and hosting intronic variants was enriched in specific GO terms in each population. Accordingly, the inputs passed to the software were the above mentioned gene list as well as a reference set, namely all genes (regardless of their known intolerance level) having intronic deletions. We focused our analysis on biological and molecular function database categories, performing the analysis with default parameters and considering as significant the associations having an FDR < 0.05.

### CNVs and GWAS catalog

We evaluated the level of association between our set of CNVs and diseases identified in the GWAS catalog (Buniello et al. 2019), using linkage disequilibrium (LD) with trait-associated SNPs as a proxy. The selected common variants underwent filtering using PLINK (version 1.9; www.cog-genomics.org/plink/1.9/) (Chang et al. 2015), removing individuals with a missing genotype rate > 0.1 and SNPs with missing call rate > 0.1, with minor allele frequency < 0.01 and those failing the Hardy–Weinberg equilibrium test. This set of filtered SNPs and our CNV set were merged together and phased using two programs, WhatsHap (version 1.1) (Patterson et al. 2015) and ShapeIt4 (version 4.1.3) (Delaneau et al. 2019), following procedures previously described (Valls-Margarit et al. 2022). The result provided the input for PLINK, where we computed LD between variants in our dataset (CNVs and SNPs) and those SNPs shared with the GWAS catalog, only including variants in high LD (*r*^2^ > 0.8) and mapping within 1 MB around the pathogenic SNP.

## Results

### Calling CNVs from whole genome sequences

We called CNVs in 40 genomes from already published Roma individuals (Bianco et al. 2020; García-Fernández et al. 2020) along with 98 samples from Europe, the Middle East and South Asia (Mallick et al. 2016; Mondal et al. 2016). Our calling pipeline comprised six programs (callers) for SV detection from WGS using GRCH38.p8 as reference genome (see “Methods” and supplementary text).

For our subsequent analyses, we included only deletions and duplications as some of the software used are unable to call insertions or inversions. We merged our data together by, first, creating a per-sample consensus among callers, finding 1484 ± 366 CNVs per sample on average (deletions: 1433 ± 352; duplications: 51 ± 23) and eventually by iteratively merging sample CNVs, obtaining calls for individuals sharing the same variant (see “Methods”). This step yielded a total number of 11,207 CNVs (9863 deletions and 1344 duplications) and an average of 1499 ± 352 CNVs per genome (deletions: 1449 ± 357; duplications: 50 ± 22).

### Dataset characteristics and population structure

We grouped our 138 samples using a geographical rationale and divided the samples as follows: Roma (40 samples), Europe (22), Middle East (15), and South Asia (61). Initially, Principal Component Analysis (PCA) revealed that samples clustered by dataset of origin (Roma, Mondal et al. (2016) and SGDP) rather than by geographic affiliation (Supplementary Fig. 1). We addressed this batch effect by regenotyping each CNV and subsequently applying different filters based on quality (allele depth, read balance in heterozygotes), checking for consistency with SNP genotypes, and Hardy–Weinberg equilibrium (see Supplementary Methods). The final filtered dataset comprised 3660 CNVs (3171 deletions and 489 duplications). We controlled for possible structure within Roma using our set of CNVs and noticed no specific relationships within regional groups (Suppl. Fig. 2 shows deletion-based analysis). In an UMAP plot based on deletions (Fig. 1), Roma individuals cluster together and apart from the Europe–Middle East–South Asia continuum; see also similar patterns for PCA (Suppl. Figs. 3, 4) and ADMIXTURE (Suppl. Fig. 5). PCA and ADMIXTURE analysis on deletion genotypes showed similar patterns to those obtained with a random SNP sample of the same size (3171, Suppl. Figs. 6 and 7). On the contrary, when applying dimensionality reduction methods to duplications (Suppl. Figs. 8, 9), this pattern was fuzzier, probably because of the higher rate and bidirectionality on mutation in duplicated segments. Thus, population history has modeled deletion (and to a lesser extent duplication) genotype frequencies in the Roma.

Out of 3660 CNVs (supplementary Fig. 10), 1899 (52%), 329 (9%) and 459 (13%) are shared by four, three and two populations respectively. We additionally found 973 (27%) variants that were found in only one population (Roma: 257, Europe: 157, Middle East: 179 and South Asia: 380), most of which were singletons. Overall, our call set is composed of 2013 common (Allele Frequency, AF) > 0.05), 668 low frequency (0.01 ≤ AF ≤  0.05) and 979 rare variants (AF < 0.01). Most common variants are shared preferentially by all four populations (four populations: 1792 (89%), three populations: 120 (6%), two populations 78 (4%), one population 23 (1%)) as expected in general populations. Low frequency variants are more evenly distributed (four populations: 107 (16%), three populations: 209 (31%), two populations 237 (36%), one population 115 (17%)) while rare variants, as expected, can be found only in one population or two at most (two populations: 144 (15%), one population: 835 (85%)) (supplementary Fig. 11). Note that these sharing proportions are underestimates, given the relatively low sample size, particularly in the Middle East. Within-population proportions of common, low frequency and rare variants change across populations, with South Asians having more variants across the frequency classes compared to the other populations and the Roma showing the same trend compared to Europe and Middle East (*χ*^2^ = 83.6, *p*-value = 6.25 × 10^–16^) (Table 1). Globally, South Asia and Roma retain a higher number of private CNVs and, evaluating the frequency profiles among populations, this pattern repeats within common, low-frequency and rare variant classes, demonstrating that the apportionment of private variants is not restricted to any specific frequency category.

**Table 1:** Distribution of CNVs for frequency class among populations

Population	*N* common	*N* low frequency	*N* rare
Roma	1967	479	288
Europe	1899	345	223
Middle East	1835	289	230
South Asia	2006	531	382

**Fig. 2:**
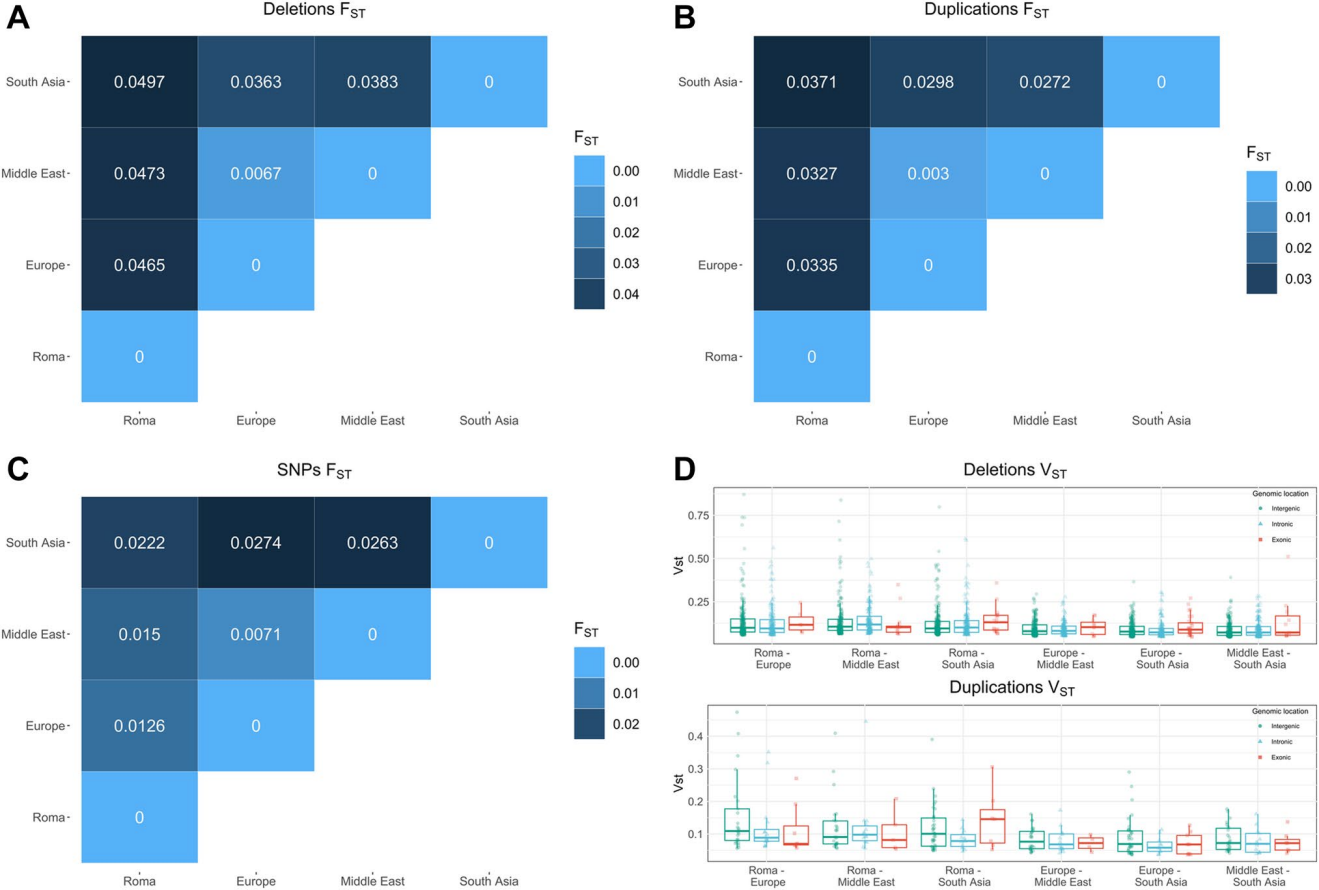
*F*_*ST*_ values for pairs of populations. For each pair of population, genome-wide Fst values are shown for deletions (**A**), duplications (**B**), and SNPs (**C**). Top quintile *V*_*ST*_ values distribution for deletions and duplications, by pairs of populations and genomic location (**D**)

Overall, the average *F*_*ST*_ (Fig. 2) among all pairs of populations was higher for deletions (0.0375) than for duplications (0.0272), which is consistent with repeat mutation at duplications counterbalancing population differentiation by drift. Thus, we will base our population inferences on deletions. The average *F*_*ST*_ between the Roma and each of the other populations was 0.0478, which is higher than for any other population. In particular, the Roma were slightly more distant from South Asia (0.0497) than from the Middle East (0.0473) or Europe (0.0465). South Asia is also equally distant from the Middle East (0.0363) and Europe (0.0383), while these two populations are close to each other (0.0067). This is the expected pattern as derived from nucleotide variation in arrays (Granot et al. 2016) or whole genomes (Mallick et al. 2016). Particularly for the Roma, these differentiation patterns are in line with previous studies based on genome-wide SNP data (Melegh et al. 2017) and could reflect the global landscape of CNVs in Roma, who had their own mutational history diverging from Northern India, ultimately admixing with Europeans and, in the process, accumulating genetic drift.

### CNV annotation

Using the software AnnotSV (Geoffroy et al. 2018, 2021) we annotated variants leveraging different databases (Refseq, OMIM, ClinGen, gnomAD, among others) and gathered information about CNV localization within genes, their possible functional role and the pathogenic consequences of their presence in transcribed genome sequences. While more than half of the CNVs in our dataset, 2115 (58%), did not overlap any currently known gene, 1532 (42%) variants intersected transcribed sequences, of which 263 (7.2%) and 1268 (35%) resided within exons and introns respectively, in agreement with previous studies (Conrad et al. 2010; Mills et al. 2011; Valls-Margarit et al. 2022) (Supplementary Fig. 12). The remaining 13 CNVs intersected more than one gene, hitting multiple intronic and/or exonic locations. Overall, we found that genomic location and the type of CNV are dependent from each other (*χ*^2^ = 77.3, *p*-value < 2.2 × 10^–16^), with deletions representing the majority of variants within each genomic location (Table 2). It is interesting to notice that exons seem to tolerate duplications better than deletions: while 6.7% of deletions affect exons, this figure is 18.2% for duplications, likely due to the stronger selective constraints over deletions within genes (Sudmant et al. 2015a). Our dataset confirms what previous studies reported about the average frequencies apportionment of intergenic and genic variants and the easier-to-resolve deletion signal used by short reads structural variants software.

**Table 2:** Number of identified deletions and duplications per genomic location. Percentages are over type of CNV

	Exonic	Intronic	Intergenic	Total
Deletions	211 (6.7%)	1111 (35.0%)	1849 (58.3%)	3171
Duplications	89 (18.2%)	134 (27.4%)	266 (54.4%)	489
Total	300	1245	2115	3660

### Geographic and genomic distribution of CNVs

**Fig. 3:**
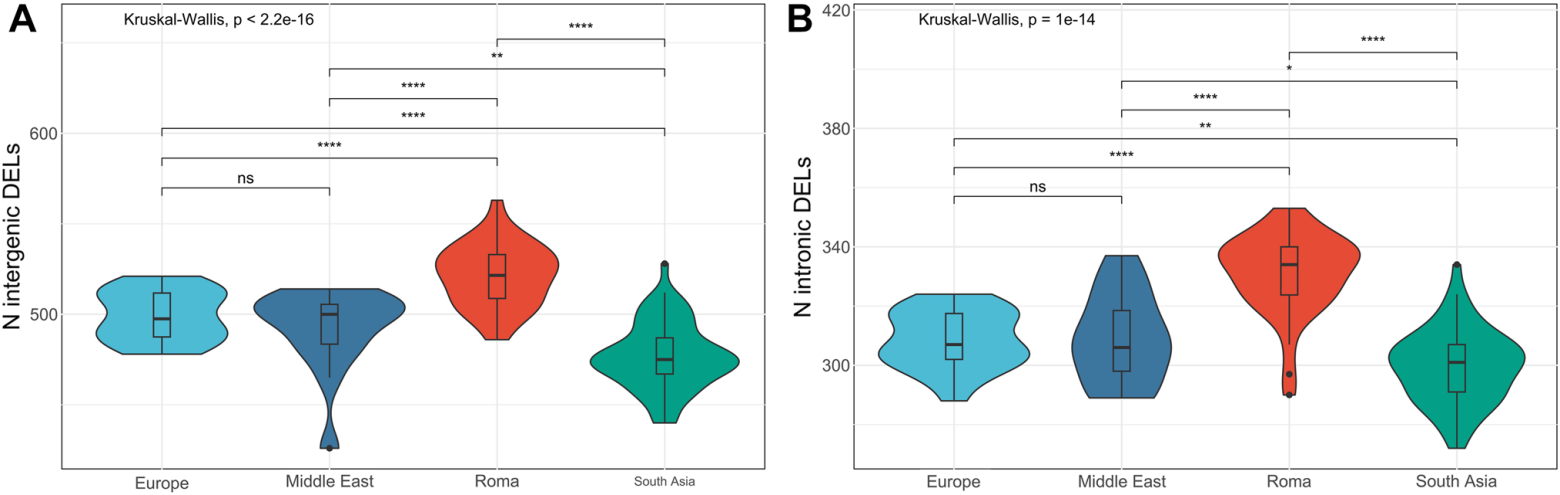
Abundance distribution and statistical tests results for deletions among populations. Statistical test and multiple comparisons results for intergenic (**A**) and intronic (**B**) deletions and their relative number distribution among populations

We next tested for the number and length of CNVs carried by individuals. For duplications, we could not find any significant difference among the populations. As for deletions, Roma carry more events per individual (mean: 880 ± 24) with respect to all other populations, (Europe: 834 ± 16; Middle East: 828 ± 29; South Asia: 810 ± 26), (Anova p-value < 2.2 × 10^–16^). Testing for deletion location, we found out that the same pattern held true for intergenic (Kruskal–Wallis p-value < 2.2 × 10^–16^) and intronic (Kruskal–Wallis *p*-value = 10^–14^) events (Fig. 3). Regarding exonic deletions, Europeans carry significantly fewer variants compared to Roma, Middle East and South Asia populations (Anova, *p*-value = 0.007). In addition, variant length also differed among populations as, overall, deletions in the Roma are larger than those in Europeans, while deletions in South Asians are shorter compared to all other populations (Kruskal–Wallis *p*-value = 1.3 × 10^–8^) (Supplementary Fig. 13A) and, within South Asia, Indian group shows shorter variants than Pakistani (Kruskal–Wallis *p*-value = 0.023). In particular, Roma have larger variants only when considering intergenic deletions, while South Asian population shows shorter intergenic, intronic and exonic (Kruskal–Wallis *p*-values, intergenic = 8.2 × 10^–10^; exonic = 0.0016 and ANOVA p-value intronic = 0.0001), (Supplementary Fig. 13). Overall, the results of these first comparisons show that Roma carry more and longer intergenic/intronic deletions than other populations, but their intolerance to exonic deletions is similar.

### Features of the highly differentiated CNVs

Next, we characterized the CNVs that were highly differentiated among populations by computing the *V*_*ST*_ statistic (Redon et al. 2006) for each CNV and pair of populations. For each variant, *V*_*ST*_ considers the variance of copy number in pairwise group comparisons; actually, for biallelic deletions (0 or 1 copies) or duplications (1 or 2 copies), *V*_*ST*_ is numerically identical to *F*_*ST*_. The mean *V*_*ST*_ values are reported in Supplementary Table 1. We focused on highly differentiated CNVs by taking the top 20% *V*_*ST*_ values, for each pair of populations (Fig. 2D); the average *V*_*ST*_ values by genomic location in this highly differentiated set can be found in Supplementary Table 2. Intergenic deletions and duplications are at the top of the value distribution; indeed, as expected, these variants display fewer constraints in the mutation rates between populations and, thus, are freer to vary. Intronic and exonic variants follow in the distribution, showing lower values for the latter calls and pointing once again to a higher constraint on those deletions and duplications putatively having a higher disruptive power over genic sequences. Since pairs containing Roma exhibited higher values at the top of the distribution, we tested if any difference existed in *V*_*ST*_ values among pairs for variants intersecting genes. We found significant differences (Kruskal–Wallis, *p*-value < 2.2 × 10^–16^) for deletions in such pairs with respect to the others. In particular, pairs considering Roma had significantly higher values than pairs without and, dividing the analysis by variant location, we could find significant differences only for intronic events (Kruskal–Wallis, *p*-value < 2.2 × 10^–16^; mean values: Roma–Europe = 0.1316; Roma–Middle East = 0.1452; Roma–South Asia = 0.131; Europe–Middle East = 0.0952; Europe–South Asia = 0.0894; Middle East–South Asia = 0.0923). Estimating variant differentiation among pairs of populations highlighted how the major source of variability can be traced back to Roma individuals; nevertheless, when stratifying the analysis by genomic location of the variants, significant differences in differentiation scores can solely be found for intronic deletions.

### Predicting the pathogenicity of CNVs

For each CNV we retrieved, whenever available, the OMIM (Online Mendelian Inheritance in Man) annotations (Hamosh et al. 2005) and the LOEUF (Loss-of-function Observed/Expected Upper Fraction) bin values (ranging in bins from 0 to 9) from gnomAD (Karczewski et al. 2020) when the variant overlapped a gene sequence. We compared the distribution of variants hitting genes having a linked OMIM entry among populations and Europeans showed a significantly lower number (Anova, *p*-value = 0.02) of deletions within OMIM genes compared to all other populations (mean number of deletions per genome: Roma = 87.8, Europe = 82.5, Middle East = 86.4, South Asia = 85.5; see supplementary Fig. 14). Duplications, instead, are significantly (Kruskal–Wallis, *p*-value = 0.007) more frequent in South Asian Indians than in South Asian Pakistani, Roma, Middle East and Europe populations (average of 7.5 for the former against 5.4, 5.7, 5.4, and 6.05 duplications per genome for the latter populations). These results could highlight a greater efficacy of natural selection removing deleterious mutations in Europeans compared to the other populations, probably due to their demographic history. Duplications within OMIM genes being more frequent in the South Asia compared to Roma and Middle East populations could reflect, to a certain degree, the increased recessive diseases specific to the group and the different selective pressures recorded for specific West Eurasian alleles, as highlighted in (Ayub and Tyler-Smith 2009; Nakatsuka et al. 2017). Roma individuals showed increased number of deletions in the 0, 1, 2 and 4 LOEUF bins and, upon stratification by location, only intronic events produced significant results for the same categories (bin 0: Anova, *p*-value = 2.8 × 10^–7^; bin 1: Kruskal–Wallis, *p*-value = 1.4 × 10^–8^; bin 2: Anova, *p*-value = 3.5 × 10^–6^; bin 4: Anova, *p*-value = 1.6 × 10^–5^). We assessed whether this higher number of deletions intersecting genes with low LOEUF values caused the overall increased number of intronic variants in Roma, as shown above. After removing these intolerant-gene deletions, Roma keep retaining a significantly higher number of intronic variants (Kruskal–Wallis, *p*-value = 5.2 × 10^–10^), demonstrating that the accumulation of these deletions at intolerant genes is an independent process that does not drive the general increase in intronic deletions.

Due to our findings of an increased number of deletions within introns of LoF-intolerant genes in Roma, we explored, separately for each population, the possibility that these mutations preferentially hit intronic coordinates while taking into account LoF tolerance. Permutation tests were performed using all genic deletions against intronic coordinates of genes either with a LOEUF ≤ 4 (intolerant) or LOEUF > 4—or for which the metrics was not available (tolerant). With these sets of regions we noticed that, while genic deletions intersect introns of tolerant genes more often than expected by chance (Permutation test, *p*-value = 0.0018–0.0004), the opposite is not true for the intersection with introns of intolerant genes (Permutation test, *p*-value > 0.05). This result points toward a general constraint for the accumulation of deletions, even at the intronic level, in intolerant genes within each population. In the context of the most differentiated variants described above, we looked at the distribution of frequencies and LOEUF values in pairwise populations containing Roma; we evaluated the frequencies in deletions showing larger differentiation, partitioning the variants across the most intolerant LOEUF classes (0–4). Despite the fact that the only significant result showed higher frequency in Roma compared to Middle Eastern population for deletions in the LOEUF 2 category (Kruskal–Wallis, *p*-value = 0.02), we noticed a general trend towards slightly higher frequencies in the Roma, across all LOEUF bins, compared to all other populations (Kruskal–Wallis, *p*-value = 0.0471). Nonetheless, pairwise group comparisons do not show significant results after multiple test correction. Following our previous results on the differentiation of intronic deletions in Roma, here we show an over-representation of such variants in this population that, together, highlight a pattern of recurring mutations occurring in untranslated genome portions. The differences in intolerant-gene deletions could highlight a lower constraint for Roma towards the accumulation of genic deletions residing outside the coding sequences but within genes whose function is more likely hampered by mutations.

### CNVs and genetic associations

In Genome-Wide Association Studies (GWAS), genetic associations are established between specific diseases or traits, or sets of them, and genetic variants, usually SNPs. We wondered to which extent the CNVs we detected could be linked to pathogenic SNPs present in the GWAS catalog (Buniello et al. 2019). To do so, we downloaded the GWAS catalog dataset version 1.0.3 and identified common SNPs between this set and those previously found in our samples (Bianco et al. 2020); the intersection consisted of 74,009 variants. For these common SNPs, we estimated the associated CNVs by selecting, for each chromosome, only those CNVs in strong linkage disequilibrium (LD) (*r*^2^ > 0.8) and residing in a 1 MB window around the SNP. Following this procedure, we identified 78 unique deletions in LD (supplementary Table 4) with 125 disease-associated SNPs as reported in the GWAS catalog, while no duplication was in linkage disequilibrium with any SNP in the set. The identified deletions are in LD with one or more (up to eight) SNPs and, for each of them, we retrieved the information about deleteriousness using LOEUF scores. Among the traits in the GWAS catalog, we could identify different functional categories. The majority of the traits involves metabolic, neurodevelopmental/neurological, development and hematological–cardiovascular disorders. Looking at the genomic context of the linked deletions, 41 (53%) reside in intergenic loci, 32 (41%) intersect introns and only five (6%) within exons. While a direct role of intergenic variants upon the pathogenicity of linked SNPs is difficult to establish—but not a reason to exclude them a priori—intronic and exonic CNVs might act on the same genomic context of the SNP. Among the intronic variants, only eight deletions intersected genes having more tolerant LOEUF scores (> 5), six other gene-intersecting variants had no score information and the remaining 18 resided in genes with higher intolerance to LoF (scores 0–4). Among these latter deletions, four are in linkage with SNPs related to metabolic/inflammatory diseases (Type 2 diabetes, alanine transaminase levels, urate levels), four others link with GWAS traits related to heart, cardiovascular or hematological conditions (myocardial infarction, hemorrhoidal disease, red-cell width) and two variants link to colorectal cancer traits. For exonic variants, only one deletion intersects an intolerant gene (LOEUF bin 4) and is in LD with a SNP associated with metabolic disorders (total cholesterol/LDL levels); nevertheless, the deletion resides in a gene upstream the SNP and its involvement is unclear. The remaining four exonic deletions associate with inflammatory diseases, lung function, hematological and developmental features and all but one (lung function) affect the same gene of the linked SNP. Nonetheless, intolerance scores are either not available or point to a relaxation against LoF for exonic variants. Finally, when considering only the set of SNPs residing ~ 5000 bp around linked deletions, we noticed that intergenic events are the most frequent type of variants in the set (19 intergenic deletions, against nine intronic and one exonic deletions). This evidence, at least in part, might support the hypothesis of a possible influence, due to physical proximity (71 bp for the closest intergenic deletion), upon the genomic environment shared with the associated pathogenic SNP. In general, using data from the GWAS catalog, we were able to leverage SNPs information as a proxy for putative CNVs involvement in health-related traits, showing that either co-occurrence of a deletion and a SNP within the same gene or physical proximity may add novel information to both the traits and to the function of the structural variant under investigation.

### Functions of the genes affected by deletions in the Roma

**Fig. 4:**
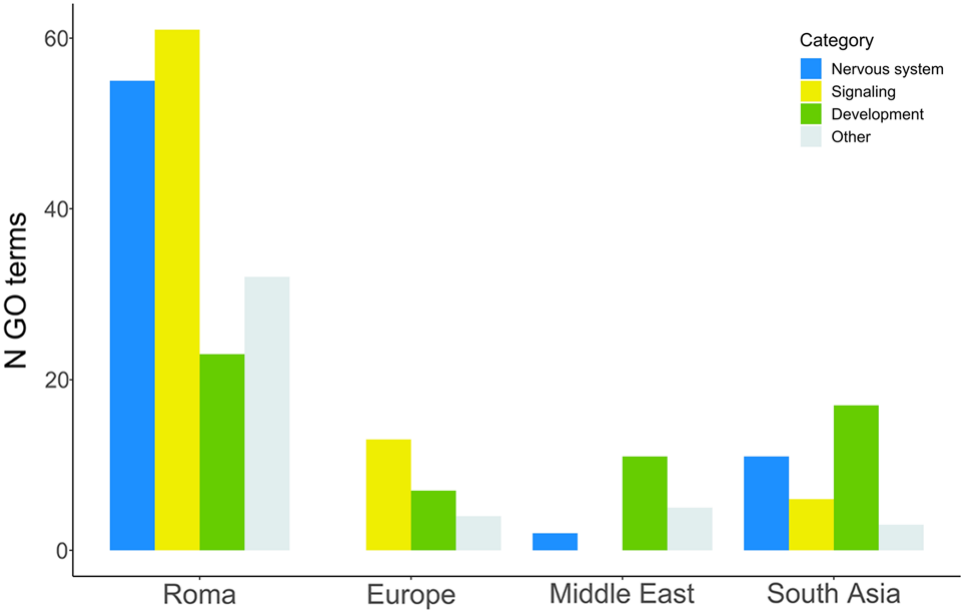
Number of categorized GO terms among populations

As previously shown, our analysis on deletion pathogenicity showed that the Roma retain a higher number of deletions intersecting LoF-intolerant genes, and specifically that intronic variants are responsible for this result. With this observation at hand, we wondered whether these more abundant intronic deletions in Roma had a specific influence on biological processes. We tested this hypothesis by performing an over-representation analysis separately in each population, using the online software GEne SeT AnaLysis Toolkit (WebGestalt) (Zhang et al. 2005; Liao et al. 2019), assessing whether LoF-intolerant genes (LOEUF bins: 0–4) intersected by intronic deletions were present more than expected in Gene Ontology (GO) terms (Ashburner et al. 2000). Results show significant enrichments in GO terms for the set of input genes in each population, with a marked prevalence of associations in Roma. Indeed, while Europe, Middle East and South Asian populations were significantly enriched for 24, 18 and 37 GO terms respectively, the Roma significant GO terms amounted to 187. For each term, using the available descriptions of related biological processes, we identified three recurrent functional categories, namely Nervous System, Signaling and Development, plus a catch-all *Other* category (Fig. 4). Overall, Roma showed higher number of GO terms among these classes compared to reference populations. The two most abundant categories in Roma were Signaling and Nervous System, which contained 61 and 55 GO terms, respectively. As a comparison, these two categories included 13/0, 0/2 and 6/11 terms in Europe, Middle East and South Asia, respectively. Furthermore, using a function within WebGestalt aiming at reducing possible redundancy for GO terms having similar gene sets, we obtained clusters of terms sharing related biological processes. Following this clusterisation, the Roma had 33 GO clusters, including 11 Signaling, 9 Nervous System, 5 Development and 8 comprising other processes such as chemotaxis, cell motility and cellular component organization. Europe, Middle East and South Asia had five, four and eight clusters with different proportions of the three major functional categories. We additionally checked for significant GO terms specifically found only in one population and noticed that Roma retain the highest number of private significant results, with 125 private terms against three, one and six found in Europeans, Middle Eastern and South Asian samples. Considering the deletions intersecting genes associated to the 125 private GO terms in Roma, we obtained 410 variants and retained only those overlapping a known pathogenic gene, either annotated in the OMIM or Deciphering Developmental Disorders (Firth and Wright 2011) (DDD) databases. The final filtered set included 168 deletions whose frequencies do not vary noticeably across populations; nevertheless, it is interesting to highlight that out of the 23 rare deletions, considering the global frequency in the whole dataset, 21 are indeed private to Roma. Within these Roma private deletions, more than half (15 variants) are singletons and reside in genes mainly associated with developmental/neurodevelopmental diseases and cancer. Of the remainder six deletions, four are doubletons and reside in genes associated to Cerebellofaciodental, Bardet-Biedl, Gillespie’s syndromes, spinocerebellar ataxia 15 and skeletal dysplasia with severe neurological disease, while the two more common variants intersect genes linked to Phelan-McDermid syndrome and -2-hydroxyglutaric aciduria. Overall, further investigation of intronic deletions in LoF-intolerant genes revealed significant enrichment in biological processes mainly related to signaling, nervous system and development, with a sharp accumulation of GO terms in Roma compared to the other populations. This supports our results of higher differentiation and abundance of intronic deletions within Roma, suggesting a possible relevance upon the functions of genes sets bearing such variants.

## Discussion

In the current study, we analyzed CNVs in the Roma population using whole genome sequencing data with the dual purpose to provide the first published catalog of genome-wide unbalanced structural variants and, given previous knowledge of Roma demographic and genetic history, assess to which extent CNVs can inform us when used in a population genetics study of an underrepresented community. Comparing deletions and duplications from Roma and other reference populations (samples from Europe, Middle East and South Asia, covering the dispersal route Roma crossed in their diaspora) we estimated the main differences in the apportionment of events, the differentiation among populations and assessed the potential biomedical impact of the variants.

### Deletions in Roma show a slight relaxation of natural selection

In our analysis, we have observed that the Roma carry more deletions than other European or Asian populations, that this additional load occurs in intergenic and intronic locations (but not in exons), that intergenic deletions in the Roma are longer, and that intronic deletions in the Roma are enriched for genes that are intolerant to loss of function (Lof) mutations. These results might be favored by population or sample-specific artifacts during deletions calling; however, variables that could affect the calling step, such as genome coverage, do not discriminate exclusively the Roma, as this latter population and the samples from SGDP share similar sequencing depth profiles. Differences in coverage among different batches, indeed, have been shown to affect CNVs calling in specific regions, but not overall (Khayat et al. 2021). Additionally, these spurious effects are unlikely to result in the apportionment observed in the Roma for deletions in introns and intergenic regions.

Although coding variation is the most obvious source of phenotypic differences, the evidence for introns and intergenic regions harboring functional variation has been accumulating (Vaz-Drago et al. 2017; Rigau et al. 2019; Telonis and Rigoutsos 2021; Keegan et al. 2022; Petersen et al. 2022). Thus, the additional intronic and intergenic deletions in the Roma point to a slight relaxation of natural selection; the effect of deletions in these regions is likely to be milder than in exons, which, in Roma, do not tolerate deletions at a higher rate than in other populations. The Roma present a unique combination of fragmentation, partial reproductive isolation, but also of admixture with their host populations. Founder events would have accumulated deletions at more tolerated locations with fewer constraints. Admixture, on the other hand, might have introduced new sources of variation in the population, while selection against deleterious mutations still acted to reduce the accumulation of harmful exonic deletions. As shown by reports on worldwide populations, usually selection acts against larger deletions in the genome (Sudmant et al. 2015a); however, in our case, this result could indicate that less efficient purifying forces may have taken place either because of the population history or because of the intergenic/intronic nature of the variants, bearing a presumably lower disrupting potential. In summary, the putative relaxed purifying selection in closed communities, which has been object of debate and addressed in finer detail by some reports (Fu et al. 2014; Balick et al. 2015; Gravel 2016; Henn et al. 2016), could be detectable only for low-impact mutations, such as intronic deletions in the Roma.

### Highly differentiated CNVs in Roma intersect some genes of biomedical interest

Estimating the differentiation for shared CNVs in pairwise population comparisons by means of *V*_*ST*_ statistics, we found that intronic variants are significantly more differentiated in pairs with Roma, driving the overall trend. We could identify only one significant frequency difference, between the Roma and Middle East populations, for intronic variants when dividing for intolerant genes categories (LOEUF bin 2), showing Roma as the population with higher frequencies. Nonetheless, we also identified a significant difference in frequencies considering all intolerant categories together (LOEUF 0–4), with Roma exhibiting higher frequencies, even though pairwise populations comparisons did not pass multiple test correction.

Exploring further the possible deleterious nature of our variants, we assessed the levels of LD with known pathogenic SNPs from the GWAS catalog and identified 78 deletions in linkage with 125 trait-associated SNPs. Out of the whole set of these associated diseases, we could highlight four categories including most conditions: metabolic, neurodevelopmental/neurological, developmental and hematological–cardiovascular disorders. Although we acknowledge that only 33 tagged deletions reside on the same gene of the associated SNP (or SNPs), most deletions (41/45) with no common gene are intergenic variants which, among all linked deletions, are those residing in closer proximity to the linked SNP(s) and, thus, might exert a specific influence on the trait-related variant. As an example, the thirty closest deletions sharing no gene with the tagged SNP are all intergenic variants and range in distance from 71 bp to 18.8 kb. This evidence points at the importance of including intergenic variants in analyses assessing CNV function, as such mutations could be either actors or co-players, modifying their genomic neighborhood, participating to different scenarios, as already reported for specific diseases (Staehling-Hampton et al. 2002; Loots et al. 2005; Farrell et al. 2011; Uyan et al. 2013). Overall, SNPs in LD with intergenic deletions show associations with traits related to development, neurodevelopmental, metabolic and hematological conditions, as well as other traits such as height, smoking behavior and heart/cardiovascular ones. For genic deletions, it is expected, and probably more likely, that their influence over gene products or regulatory functions would be stronger than intergenic ones. Together, this set of deletions primarily associate to metabolic/inflammatory, cancer and neurodevelopmental/neurological traits. The collection of conditions related to metabolism mainly pertains to cholesterol levels, type 2 diabetes, alanine transaminase levels and obesity traits. Genes containing SNPs in LD with deletions had low reported LOEUF values, indicating their intolerance to loss of function (*CCDC50*, *JAZF1*, *MYO9A*, *CNOT1* genes having, respectively, three, one, one and zero LOEUF bin scores). Intriguingly, a previous study showed how European Roma carried higher frequencies of SNPs involved in hyperlipidemia (Mendizabal et al. 2013); we found one deletion in *RHCE* gene in linkage with one cholesterol-associated SNP within the neighboring *MACO1* gene (however, a direct functional effect upon the *RHCE* gene, which codes for a Rh-like red blood cell antigen, should not be dismissed), and indeed the deletion is higher in frequency within Roma.

### Genes intersected by CNVs in Roma are enriched for central nervous system functions

We discovered that Roma carry a marked prevalence of GO terms associated to common functions subsets of inputted genes lists. Intriguingly, we could highlight marked differences only when using this type of gene sets, i.e., intolerant genes that contained intronic deletions, and not while using other sets, such as private deletions within populations or general classification based on genomic location. This is unlikely the result of a general higher number of deletions in Roma but rather the specific function of the affected genes. Roma show more biological process GO terms in each defined category (Nervous system, Signaling and Development categories plus “Other” containing general unrelated terms) compared to the other populations, and a strong difference can be noticed for the Nervous system and Signaling categories. We find these results of particular interest in light of the known private diseases specifically affecting Roma people in Europe. Indeed, among the different types of private disease-causing mutations described in the Roma, some involve neuropathies and neurological diseases such as hereditary motor and sensory neuropathy-Lom/Russe types, congenital cataracts facial dysmorphism neuropathy and limb-girdle muscular dystrophy type 2C (Kalaydjieva et al. 1996, 2001, 2005; Angelicheva et al. 1999; Morar et al. 2004). Nervous system-related GO terms often involved neurons connections organization, synaptic communication or brain development, highlighting the presence of putatively deleterious variants affecting physiological neuronal functions particularly in Roma, in line with previous reports of a higher rate of slightly deleterious variants, for other disorders, in Roma individuals (Mendizabal et al. 2013). Two examples of private intronic deletions in Roma (Supplementary Fig. 15), intersecting LoF genes implicated in central nervous system functions are in gene *WDPCP* (LOEUF score: 4), with GO terms related to CNS, such as “neuron differentiation”, “cell projection organization”, “cell morphogenesis involved in neuron differentiation”, “neuron development” and gene *SHANK3* (LOEUF score: 0) with GO terms description include, among others, “regulation of nervous system development”, “telencephalon development”, “synaptic signaling”, “axongenesis”. Moreover, the disease-associated SNPs assessed in (Mendizabal et al. 2013) reside in genes belonging to biological processes associated to the significant GO terms we identified in our analysis, highlighting a possible action of different markers (deletions and SNPs) within same sets of genes, specifically affecting their functions. Lastly, as a general point, it is important to bear in mind that frequency spectra for clinically relevant variants differ among populations, with Roma showing their relatively low isolation by exhibiting highly represented deleterious alleles as well as near absence of others, whose ancestries are mainly related to South Asian and European haplotypes (Font-Porterias et al. 2021).

### Isolated populations are an under-analyzed genomic resource, also for CNVs

Populations of non-European descent have traditionally been understudied in the context of genetic variation, particularly favoring GWAS research on more accessible cohorts of general European ancestry (Bustamante et al. 2011; Popejoy and Fullerton 2016). Ironically, what should be one important goal of human genetics research: uncovering an increasingly clearer and more complete picture of human genetic variation worldwide, portray a fairer representation of different human populations and advancing current knowledge on genetic diseases using diverse sets of populations (Zeggini 2014), has often been disregarded in favor of a Eurocentric perspective (Need and Goldstein 2009; Sirugo et al. 2019). Numerous studies addressing population isolates, indeed, contributed significantly to identify the loci underlying complex diseases: bipolar disorder and schizophrenia in Finland and Basque populations (Palo et al. 2007; Parsons et al. 2007), studies on Iceland individuals highlighting variants associated to atrial fibrillation, myocardial infarction, type 2 diabetes and glaucoma (Manolescu et al. 2004; Gudbjartsson et al. 2007; Helgadottir et al. 2007; Steinthorsdottir et al. 2007; Thorleifsson et al. 2007) and also traits as height and pigmentation in Finland, Iceland, Sardinia and Amish populations (Sulem et al. 2007, 2008; Gudbjartsson et al. 2008; Sanna et al. 2008). It has been suggested that addressing isolated populations for studying diseases can help in reducing the variance of environmental variables on pathogenic conditions, as homogeneity in phenotype and environment within isolates would facilitate the disease–gene recognition (Kristiansson et al. 2008), thus favoring the inclusion of underrepresented populations to advance our understating of health-related traits.

## Supplementary Information

Below is the link to the electronic supplementary material.

Supplementary file1 (DOCX 266 KB)

Supplementary file2 (DOCX 3533 KB)

Supplementary file3 (XLSX 29 KB)

## Data Availability

Upon acceptance of this manuscript, a vcf file containing the CNV calls for the samples analyzed will be deposited in the European Genome-Phenome Archive (EGA). In-house scripts used in this work can be retrieved from https://github.com/marcoantinucci/CNVs_scripts.
