# Supplementary material for: Population history modulates the fitness effects of Copy Number Variation in the Roma
Source: Hum Genet. 2023 Jun 14;142(9):1327–43. doi: 10.1007/s00439-023-02579-5 (PMC10449987; doi:10.1007/s00439-023-02579-5)
Supplement: Supplementary file 1 — Supplementary file1 (DOCX 266 KB) [file 439_2023_2579_MOESM1_ESM.docx]

**Supplementary materials**

**Population history modulates the fitness effects of Copy Number Variation in the Roma**

Marco Antinucci, David Comas, Francesc Calafell(*)

Institute of Evolutionary Biology (UPF-CSIC), Department of Medicine and Life Sciences, Universitat Pompeu Fabra, Barcelona, Spain

* Corresponding author

*Sequencing methods of the original datasets*

For Roma samples (Bianco et al. 2020), DNA molecules from samples were fragmented and adapter ligated. The fragments with adapter were then amplified via PCR and subsequently gel purified. Sequencing was performed using Illumina HiSeq X to produce 150bp paired-end reads. Experiments were carried out in a single location. Samples average coverage is ~30X.

For the Mondal et al. (2016) samples, blood (most) and saliva samples sequenced at Beijing Genomics Institute (BGI) and National Institute of Biomedical Genomics (NIBG, India). At BGI, 1µg of genomic DNA was sheared with Covaris E210 system (CovarisInc). DNA fragments sticky ends were blunted with T4 DNA polymerase and Klenow enzyme. After selection of ~500bp fragments with gel electrophoresis and subsequent purification, PCR was used to amplify these fragments. Sequencing was carried out via Illumina HiSeq 2000 to produce 90bp paired-end reads. At NIGB, 1µg of genomic DNA was sheared by Covaris (CovarisInc) 3-400bp fragments. Fragments ends were repaired by mixing with End Repair Mix (TruSeq DNA Sample Prep Kit, Illumina) and purified using Ampure XP (Beckman Coulter). Subsequently, libraries were 3’-adenylated and ligated to DNA Adapter Indexes, followed by further purification via Ampure XP system (Beckman Coulter) and gel electrophoresis. Fragments were PCR-amplified and their quality and quantity were assessed in a High Sensitivity DNA chip in 2100 Bioanalyzer (Agilent) and by Real Time PCR with Kapa Library Quant Kit (Kapa Biosystems) in ABI 7900HT system (Life Technologies). Fragments of 4-500bp and yield of 500ng were then sequenced with Illumina HiSeq 2000 System producing 100pb paired-end reads. The sample average coverage was ~12X

Finally, for the SGDP samples Mallick et al. (2016) about 2.5 µg of genomic DNA for every sample was submitted to Illumina Ltd for standard high coverage sequencing; library preparation did not include a PCR amplification step. Samples were sequenced on a HiSeq2000 sequencer producing paired-end reads of 100bp in length, insert length distribution was 314 ± 20 bases. All samples were sequenced the same day to control for potential artificial differences among samples. Sample average coverage is ~35X

*Structural variant calling algorithms*

Different methods exist to call structural variants (SVs) from short read sequencing data. To infer the presence of structural sequence changes in a sample (or a group of samples), a number of methodologies leverage in different ways the information from mapped sequences. The most frequently used algorithms devised so far are based on read depth, split reads, read pairs or assembly approaches. Briefly, read-depth method assumes that the coverage of a region relates to the number of copies of that region. To assess this, the method counts the number of reads mapping to fixed size regions (bins) and, after data normalization, estimates the number of copies in the genome (Tattini et al. 2015). The split-read approach takes advantage of how paired-end reads map: if one of the paired reads accurately maps to the reference while the other does not or only maps partially, the latter may signal the presence of a SV breakpoint. The splitting of the unmapped (or partially mapped) read in sub-reads allows for a second mapping step of these portions independently. After this step, the two portions of the split read will flank respectively the start and end point of the detected SV (Zhao et al. 2013). In the read-pair approach, SVs are detected by relying on the spacing between read pairs mapped to reference. Pairs of reads mapping closer or further to one another than what is expected based on their average insert size signal the presence of an SV (Zhao et al. 2013). The last method, assembly-based (or *de novo* assembly), uses groups of overlapping reads to create contigs; these sequences are longer than short reads and represent the union of non-repetitive information that the latter provide. The comparisons between contigs and a reference genome highlight regions with putative discordant copy number, where SV may occur (Tattini et al. 2015). Here we provide the list of software used to call SVs and the settings we used.

*CNVnator*

We ran CNVnator (version 0.4.1) (Abyzov et al. 2011) using a bin size of 100bp, identifying duplication and deletion calls for each sample. Results then underwent copy number estimation using the –genotype command and the results were filtered for calls having e-val1, e-val2 and q0 parameters with values lower than 0.05, 0.005 and 0.5 respectively.

*BreakDancer*

As a first step using the BreakDancer (version 1.4.5) pipeline (Chen et al. 2009), we used the built-in script bam2cfg.pl to obtain relevant data from the input BAM files, such as read length, average insert size and standard deviation, to be used subsequently by the algorithm. We then checked for a number of parameters (i.e. RG or LB information in the header, coefficient of variation of the insert size, percentage of inter-chromosomal read pairs) to assess the quality of the input files. BreakDancer with default parameters generates raw results, subsequently filtered to retain copy numbers equals to zero, one, three or more. For further refinement of the results, we implemented the Perl BreakDown software (version 1.1.1) (Fan et al. 2014) to work with the BreakDancer output, obtaining genotype calls and filtered results. We turned on GC correction (-g option), and filtered for variant score lower than 40, for mapping quality lower than 30 (-q option) and for event size lower than 100bp.

*Pindel*

Pindel (version 0.2.5b8) (Ye et al. 2009) uses the split-read method, and we ran it with default parameters, including BreakDancer result calls as a support to increase its sensitivity and specificity. Once the main script was executed, we applied filters removing those calls shorter than 100bp, or having a mapping quality lower than 30 and a number of supporting reads lower than 20% the average coverage of the samples included in the analysis.

*Tardis*

We first used mrsFAST (version 3.4.0) (Hach et al. 2014) to extract discordant read pairs from the paired-end reads (fastq) files of each sample, providing the read-pair information for subsequent analysis. Discordant reads refer to read pairs spacing outside the minimum-maximum range of the fragment size and mapping respectively to forward and reverse strand. We ran Tardis (version 1.0.4) (Soylev et al. 2017) with default parameters and we excluded variants below 100bp.

*Lumpy*

To prepare support data for Lumpy (version 0.2.13) (Layer et al. 2014), we used SpeedSeq (version 0.1.2) (Chiang et al. 2015), which takes fastq files as input to generate splitters and discordant files reporting information about split-read and read-pair methods. Lumpy also integrates CNVnator results for further read-depth information. We ran the software with default parameters and excluding low complexity regions of the genome that may lead to unreliable results. The output underwent the SVTyper (version 0.7.1) (Chiang et al. 2015) algorithm to produce genotyped results. We filtered out calls with mapping quality lower than 30, a number of supporting reads lower than 5 and size lower than 100bp.

*GenomeSTRiP*

We used the GenomeSTRiP (version 2.00.1918) (Handsaker et al. 2011; Handsaker et al. 2015), which implements the CNVDsicovery pipeline. We first performed data pre-processing with the SVPreprocess pipeline, which generates metadata needed for all the subsequent steps. We then applied the CNVDsicovery pipeline, to call for CNVs in our sample set and finally run SVGenotyper pipeline to obtain genotyped calls for our results. We finally removed those variants having low quality scores (CNQ > 12; “LQ”) from further analyses and events smaller than 100bp.

Average CNVs called per genome for each software. DEL: deletions, DUP: duplications, INS: insertions, INV: inversions, RD: read-depth, RP: read-pair, SR: split-read

|  | Average counts per genome | | | | |
| --- | --- | --- | --- | --- | --- |
| **Software** | **Total SVs** | **DEL** | **DUP** | **INS** | **INV** |
| CNVnator (RD) | 1753 | 1356 | 397 | - | - |
| BreakDancer (RP) | 2784 | 2717 | - | 62 | 5 |
| Pindel (SR) | 1184 | 1059 | 114 | 11 | - |
| Tardis (SR, RD, RP) | 822 | 650 | 114 | 7 | 40 |
| Lumpy (RP, SR) | 2290 | 1974 | 243 | - | 73 |
| GenomeSTRiP (SR, RD, RP) | 1893 | 1525 | 368 | - | - |

*CNV caller performance*

As a measure of the performance of each CNV caller, we considered for each individual how many CNVs were called by 2,..,6 different callers, both in absolute numbers (panel A below) and as the proportion of the CNVs called (panel B). As can be seen in the figure, Lumpy was overrepresented in the calls confirmed by other software. Only 20 CNVs were called by all programs, and since, obviously, all six achieved 100% precision in that set, we omitted it from plot B below.

*
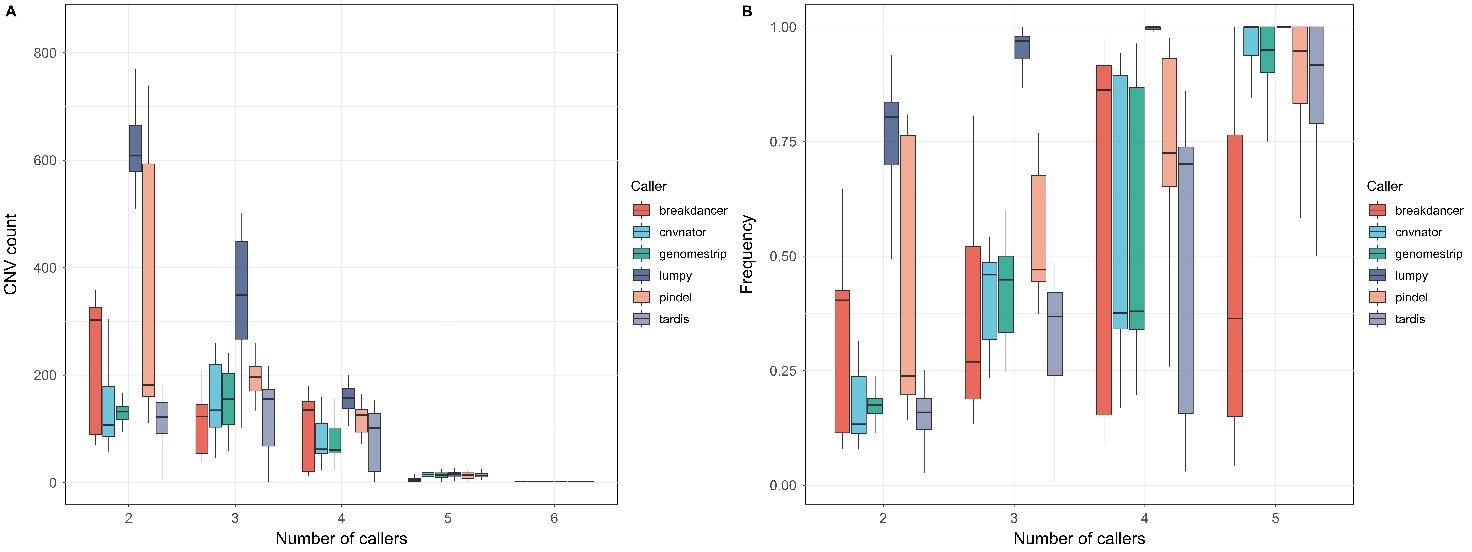
*

*Batch effect filtering*

When using PCA on copy numbers in deletions and duplications, our dataset showed batch effects, clustering the samples based on the dataset they belong to. We attempted to overcome batch effects by refining our genotype calls for deletions and duplications and by subsequently filtering the resulting set of variants. We specifically re-estimated each CNV genotype for all the individuals in our dataset by using GraphTyper2 (version 2.5.1) (Eggertsson et al. 2019). We retained only variants called with the “AGGREGATED” model and used the subsequent command line from vcffilter software:

vcffilter -f "( SVTYPE = DEL & QD > 12 & ( ABHet > 0.30 | ABHet < 0 ) & PASS_AC > 0 & PASS_ratio > 0.1 ) | ( SVTYPE = DUP & QD > 5 & PASS_AC > 0 )"

This filter includes deletions with a ratio of quality call/alt allele depth higher than 12, the allele balance for heterozygous calls (read count of call2/(call1+call2), which is -1 if there’s no heterozygous call) higher than 0.3 or lower than 0, having at least 1 called genotypes having “PASS” filter and at least 0.1 ratio of PASS called genotypes among samples. For duplications it requires a quality call/alt allele depth higher than 5 and at least one called genotypes having “PASS” filter.

After quality filtering, we accurately recovered 7257 variants (6070 deletions and 1187 duplications), filtering out ~35% of the variants present in the initial set. Since a portion of the variants detected were false positives (i.e. initially detected as heterozygous/homozygous for a CNV and subsequently genotyped as homozygous for the reference allele), a deeper scan for such calls was conducted using the R packages CNVfilteR (version 1.8.0) (Moreno-Cabrera et al. 2021) and HardyWeinberg (version 1.7.2) (Graffelman 2015). The former uses SNP data to identify incorrectly called deletions and duplications, while the latter performs chi-squared test for Hardy-Weinberg equilibrium. We filtered out 778 (520 deletions and 258 duplications) and 2819 (2379 deletions and 440 duplications) variants using CNVfilteR and HardyWeinberg packages respectively. The final filtered dataset comprised 3660 CNVs (3171 deletions and 489 duplications). The larger number of deletions compared to duplications probably reflects the higher performance of software to resolve deletion events compared to duplications (Zook et al. 2020; Khayat et al. 2021).

*Data merging*

We designed custom scripts to obtain the data both for the results for all callers for a single sample and among all samples. To do so, we first merged the output of the different software for each sample, specifically by merging those SVs residing on the same chromosome, deletions and duplications separately, with a reciprocal genomic coordinate overlap of at least 50% of their length. By doing so, we created clusters of overlapping pairs of calls and for each cluster (ranging from a pair of calls for two programmes, up to 15 pairs, corresponding to all possible combination of pairs –without self-pairing- among the six software used) we selected the coordinates and the genotype of the most confident caller, based on the evaluation of caller performance in (Kosugi et al. 2019). For deletions coordinates we used the following hierarchy: 1) lumpy, 2) pindel, 3) genomestrip, 4) breakdancer, 5) tardis, 6) cnvnator. Duplication coordinates, instead, respected the following hierarchy: 1) pindel, 2) lumpy, 3) cnvnator, 4) genomestrip, 5) tardis, 6) breakdancer.

Both deletions and duplications followed the same genotyped hierarchy: 1) cnvnator, 2) lumpy, 3) genomestrip, 4) tardis, 5) breakdancer, 6) pindel.

Using this information for each cluster of calls mentioned above, we obtained a single call by retaining the best performing software for coordinates and genotype respectively. To merge variants across samples we proceeded in a similar manner as previously presented, where we joined all sample calls if variants of the same type resided on the same chromosome and reciprocally overlapped at least for 50% of their length. This allowed us to create a consensus set of calls listing the sharing of each variant among individuals.

*Benchmark of CNV calling pipeline*

In order to test the accuracy of our CNV calling pipeline, we tested our procedure on a gold standard sample from Genome In a Bottle (GIAB) Consortium (Zook et al. 2016). We downloaded the bam, fastq and SVs true set files for the European HG001/NA12878 sample. As the data were generated at 300X, we first downsampled them to ~30X to better represent our dataset and using the mapped and row reads files we implemented our set of callers for CNV detection. We managed to use all but one CNV calling software, GenomeSTRiP, which is designed for population-scale calling. Efforts to overcome its inclusion were unsuccessful and forced us to exclude GenomeSTRiP for the calling validation. We merged the different caller results using our pipeline to obtain 2009 deletions, 199 duplications and 20 inversions.

To compare our results to the true set available for HG001, SVs true by Mohiyuddin et al. (2015) set was lift to the hg38 version of the genome to match the coordinates we used for the calling phase. We than used the script witty.er (<https://github.com/Illumina/witty.er#cross-type-matching-between-cnv-and-svdirectional-matching-for-cnv>) to compare overlapping calls between a query set against a true set of SVs. The validation results returned a total of 1518 deletions, detected by our pipeline and shared with the true positive set, which implies a precision of 76.44% and a recall of 58.5%, with an overall F-score of 66.28%. We also merged the true SV set to another available for HG001 sequenced using PacBio technologies and the results were 82.23% precision, 40.69% recall and 54,44% F-score.

*CNVs and Topologically Associated Domains (TADs)*

We annotated CNVs intersecting Topologically Associated Domains (TADs). TADs are defined as genome portions in close physical contact due to the three-dimensional arrangement of DNA sequences, which are more likely to interact with one another than with adjacent sequences. We assessed the degree of overlap between CNVs and TADs and identified eight variants (five deletions and three duplications, Supplementary Table 3), of which two singletons, intersecting these regions and at least one gene. Among our findings, we noticed interesting examples of deletions at clinically relevant genes, such as a 183kb deletion which affected both the first intron of *ADGRL4*, a gene involved in angiogenesis, and a TAD (coordinates: 1:79254316-80254315) found in the initial portion of the gene. Another deletion completely removes a gene involved in steroid metabolism, *UGT2B28*, and part of a TAD sequence encompassing other related genes (*UGT2B4* and *UGT2A2*). Furthermore, an intriguing example involves a 151kb deletion spanning five immune system-related genes (*IGHG4*, *IGHG2*, *IGHA1*, *IGHG1* and *IGHG3*) on the telomeric portion of chromosome 14. The three most distal genes are also contained in an extended TAD sequence (coordinates: 14:105653664-106053818) including a dense cluster of immunoglobulin genes. In these examples, the frequency of the deletions is evenly distributed among the populations sharing the variant, except for the latter, where Roma show the highest (0.313) within an increasing frequency cline from South Asia (0.107) to Middle East (0.167) to Europe (0.205). It is also worth noticing that all the TAD-intersecting variants share a same common feature: their relatively large size. Indeed, the five deletions have a mean size of 203kb (compare to the overall 4kb mean for deletions), while duplication average length is 193kb (as opposed to their 9.4kb mean size across the genome). This may be just a spurious effect: longer CNVs may be more likely to intersect TADs or any other genomic feature just because they are longer.

Duplications are either singletons/rare events (such as a duplicated sequence overlapping *GRHL2* transcription factor in 3 individuals), or widespread (present in 78 samples mainly intersecting *NBPF1* a member of a highly duplicated family (Vandepoele et al. 2005)). Together, these findings provide hints for 3D genome conformations whose function - typically fostering gene transcription by facilitating the action of enhancers upon gene promoters (Beagan and Phillips-Cremins 2020) - might be impaired or disrupted by deletion events, particularly when multiple genes encoding similar products reside closer together (Lupiáñez et al. 2016; Shanta et al. 2020).

**References**

Abyzov A, Urban AE, Snyder M, Gerstein M. 2011. CNVnator: An Approach to Discover, Genotype and Characterize Typical and Atypical CNVs from Family and Population Genome Sequencing. :974–984.

Beagan JA, Phillips-Cremins JE. 2020. On the existence and functionality of topologically associating domains. *Nat Genet* 52:8–16.

Bianco E, Laval G, Font-Porterias N, García-Fernández C, Dobon B, Sabido-Vera R, Sukarova Stefanovska E, Kučinskas V, Makukh H, Pamjav H, et al. 2020. Recent common origin, reduced population size, and marked admixture have shaped European roma genomes. *Mol Biol Evol* 37:3175–3187.

Chen K, Wallis JW, McLellan MD, Larson DE, Kalicki JM, Pohl CS, McGrath SD, Wendl MC, Zhang Q, Locke DP, et al. 2009. BreakDancer: An algorithm for high-resolution mapping of genomic structural variation. *Nat Methods* 6:677–681.

Chiang C, Layer RM, Faust GG, Lindberg MR, Rose DB, Garrison EP, Marth GT, Quinlan AR, Hall IM. 2015. SpeedSeq: Ultra-fast personal genome analysis and interpretation. *Nat Methods* 12:966–968.

Eggertsson HP, Kristmundsdottir S, Beyter D, Jonsson H, Skuladottir A, Hardarson MT, Gudbjartsson DF, Stefansson K, Halldorsson B v., Melsted P. 2019. GraphTyper2 enables population-scale genotyping of structural variation using pangenome graphs. *Nat Commun* [Internet] 10:1–8. Available from: http://dx.doi.org/10.1038/s41467-019-13341-9

Fan X, Zhou W, Chong Z, Nakhleh L, Chen K. 2014. Towards accurate characterization of clonal heterogeneity based on structural variation. *BMC Bioinformatics* 15:1–12.

Graffelman J. 2015. Exploring diallelic genetic markers: The HardyWeinberg package. *J Stat Softw* 64:1–23.

Hach F, Sarrafi I, Hormozdiari F, Alkan C, Eichler EE, Sahinalp SC. 2014. MrsFAST-Ultra: A compact, SNP-aware mapper for high performance sequencing applications. *Nucleic Acids Res* 42:494–500.

Handsaker RE, Van Doren V, Berman JR, Genovese G, Kashin S, Boettger LM, Mccarroll SA. 2015. Large multiallelic copy number variations in humans. *Nat Genet* 47:296–303.

Handsaker RE, Korn JM, Nemesh J, McCarroll SA. 2011. Discovery and genotyping of genome structural polymorphism by sequencing on a population scale. *Nat Genet* 43:269–276.

Khayat MM, Mohammad S, Sahraeian E, Zarate S, Carroll A, Hong H, Pan B, Shi L, Gibbs RA, Mohiyuddin M, et al. 2021. Hidden biases in germline structural variant detection. *Genome Biol* 22:347.

Kosugi S, Momozawa Y, Liu X, Terao C, Kubo M, Kamatani Y. 2019. Comprehensive evaluation of structural variation detection algorithms for whole genome sequencing. *Genome Biol* 20:8–11.

Layer RM, Chiang C, Quinlan AR, Hall IM. 2014. LUMPY: A probabilistic framework for structural variant discovery. *Genome Biol* 15:1–19.

Lupiáñez DG, Spielmann M, Mundlos S. 2016. Breaking TADs: How Alterations of Chromatin Domains Result in Disease. *Trends in Genetics* 32:225–237.

Mallick S, Li H, Lipson M, Mathieson I, Gymrek M, Racimo F, Zhao M, Chennagiri N, Nordenfelt S, Tandon A, et al. 2016. The Simons Genome Diversity Project: 300 genomes from 142 diverse populations. *Nature* 538:201–206.

Mohiyuddin M, Mu JC, Li J, Bani Asadi N, Gerstein MB, Abyzov A, Wong WH, Lam HYK. 2015. MetaSV: An accurate and integrative structural-variant caller for next generation sequencing. *Bioinformatics* 31:2741–2744.

Mondal M, Casals F, Xu T, Dall’Olio GM, Pybus M, Netea MG, Comas D, Laayouni H, Li Q, Majumder PP, et al. 2016. Genomic analysis of Andamanese provides insights into ancient human migration into Asia and adaptation. *Nat Genet* [Internet] 48:1066–1070. Available from: http://dx.doi.org/10.1038/ng.3621

Moreno-Cabrera JM, del Valle J, Castellanos E, Feliubadaló L, Pineda M, Serra E, Capellá G, Lázaro C, Gel B. 2021. CNVfilteR: an R/Bioconductor package to identify false positives produced by germline NGS CNV detection tools. *Bioinformatics*:1–3.

Shanta O, Noor A, Chaisson MJP, Sanders AD, Zhao X, Malhotra A, Porubsky D, Rausch T, Gardner EJ, Rodriguez OL, et al. 2020. The effects of common structural variants on 3D chromatin structure. *BMC Genomics* 21:1–10.

Soylev A, Kockan C, Hormozdiari F, Alkan C. 2017. Toolkit for automated and rapid discovery of structural variants. *Methods* 129:3–7.

Tattini L, D’Aurizio R, Magi A. 2015. Detection of Genomic Structural Variants from Next-Generation Sequencing Data. *Front Bioeng Biotechnol* [Internet] 3:1–8. Available from: http://journal.frontiersin.org/Article/10.3389/fbioe.2015.00092/abstract

Vandepoele K, Van Roy N, Staes K, Speleman F, Van Roy F. 2005. A novel gene family NBPF: Intricate structure generated by gene duplications during primate evolution. *Mol Biol Evol* 22:2265–2274.

Ye K, Schulz MH, Long Q, Apweiler R, Ning Z. 2009. Pindel: A pattern growth approach to detect break points of large deletions and medium sized insertions from paired-end short reads. *Bioinformatics* 25:2865–2871.

Zhao M, Wang Qingguo, Wang Quan, Jia P, Zhao Z. 2013. Computational tools for copy number variation (CNV) detection using next-generation sequencing data: Features and perspectives. *BMC Bioinformatics* [Internet] 14:S1. Available from: http://www.biomedcentral.com/1471-2105/14/S11/S1

Zook JM, Catoe D, McDaniel J, Vang L, Spies N, Sidow A, Weng Z, Liu Y, Mason CE, Alexander N, et al. 2016. Extensive sequencing of seven human genomes to characterize benchmark reference materials. *Scientific Data 2016 3:1* [Internet] 3:1–26. Available from: https://www.nature.com/articles/sdata201625

Zook JM, Hansen NF, Olson ND, Chapman L, Mullikin JC, Xiao C, Sherry S, Koren S, Phillippy AM, Boutros PC, et al. 2020. A robust benchmark for detection of germline large deletions and insertions. *Nat Biotechnol* [Internet] 38:1347–1355. Available from: http://dx.doi.org/10.1038/s41587-020-0538-8
