# Supplementary material for: Population history modulates the fitness effects of Copy Number Variation in the Roma
Source: Hum Genet. 2023 Jun 14;142(9):1327–43. doi: 10.1007/s00439-023-02579-5 (PMC10449987; doi:10.1007/s00439-023-02579-5)

**Supplementary Figures**

**Population history modulates the fitness effects of Copy Number Variation in the Roma**

Marco Antinucci, David Comas, Francesc Calafell

Institute of Evolutionary Biology (UPF-CSIC), Department of Medicine and Life Sciences, Universitat Pompeu Fabra, Barcelona, Spain

Address correspondence to Francesc Calafell, [francesc.calafell@upf.edu](mailto:francesc.calafell@upf.edu)

Supplementary Figure 1 - PCA of the unfiltered dataset. Batch effect in the dataset. PCA plots at the top (A and B) show analysis with deletions, bottom plots (C and D) show duplications. Points shape and colour follow population (A and C) and dataset (B and D) labels


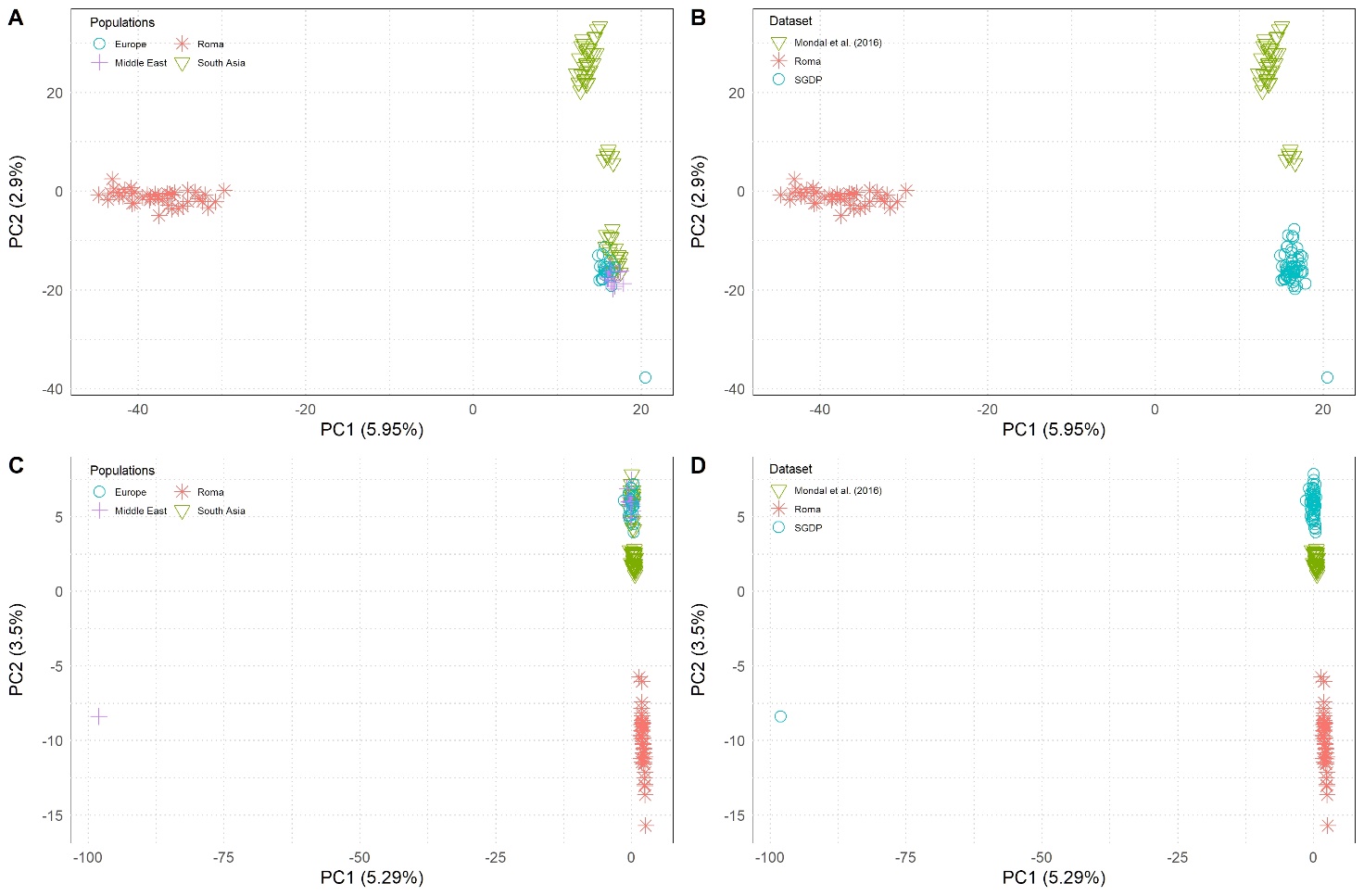


Supplementary Figure 2 - PCA of regenotyped and filtered dataset deletions. Analysis performed on Roma samples using geographical labels.


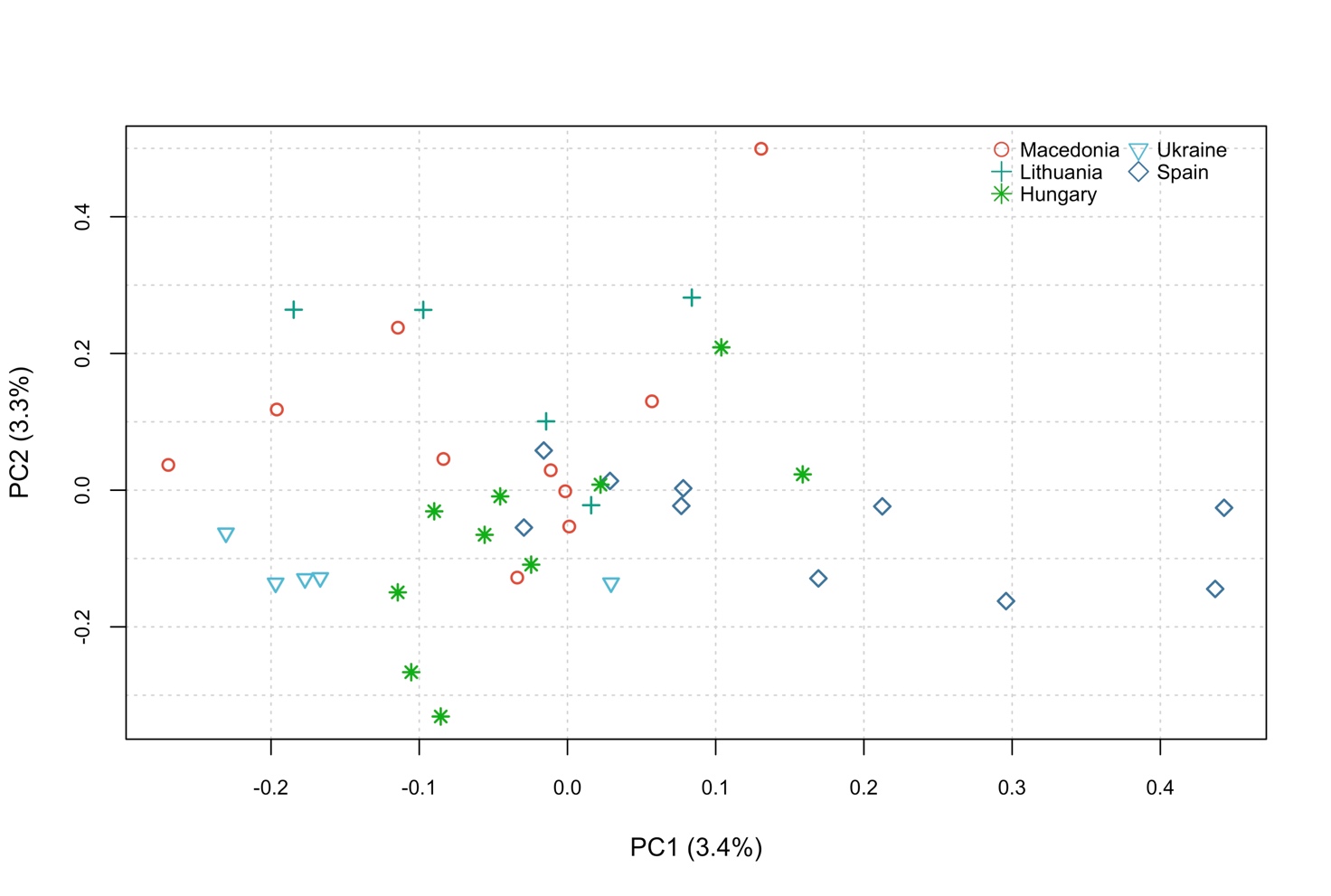


Supplementary Figure 3 - PCA of regenotyped and filtered dataset deletions. PCA plots with population (A, C) and dataset (B, D) labels. Upper plots show principal components 1 and 2, while lower plots show principal components 3 and 4.


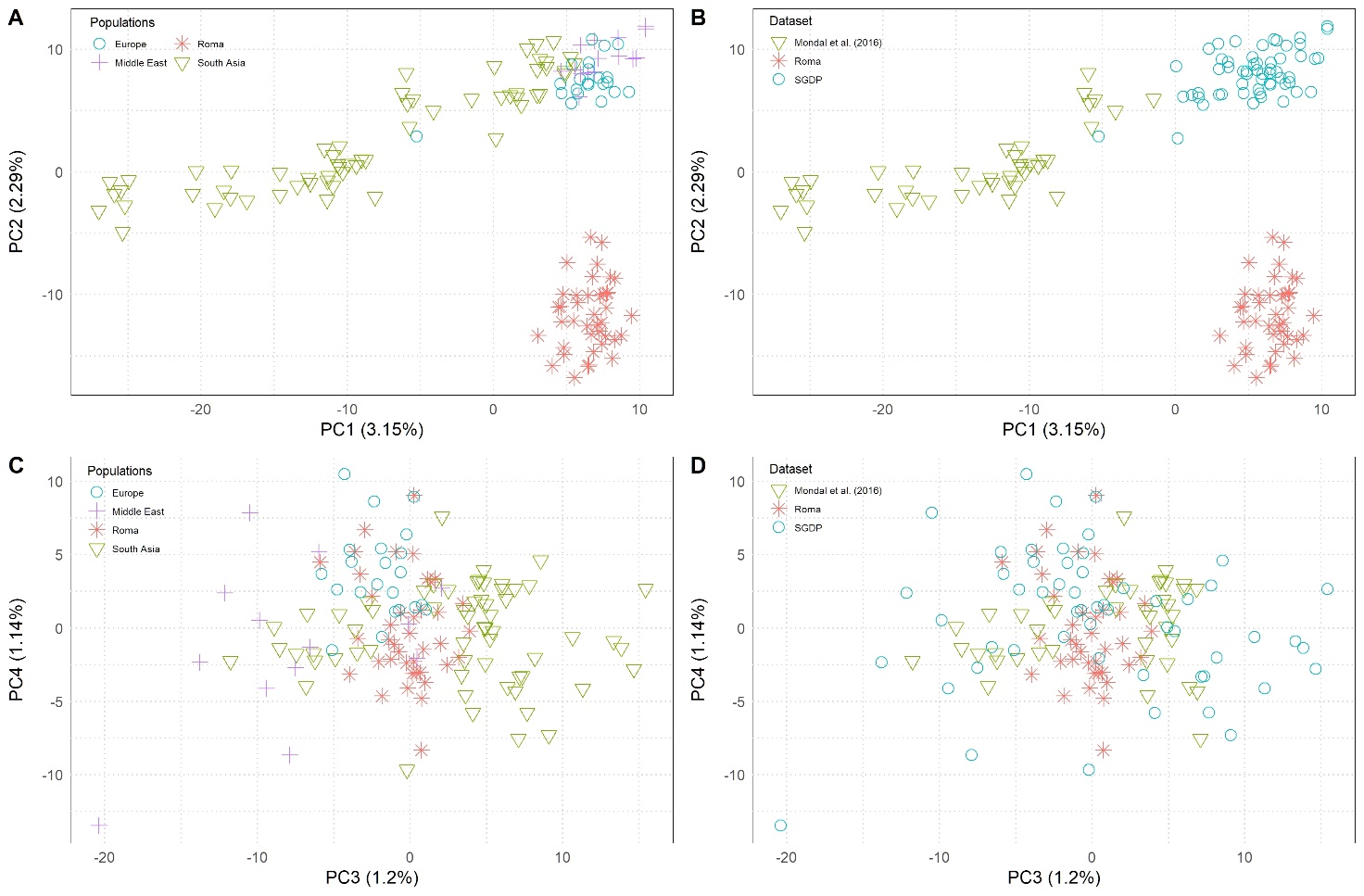


Supplementary Figure 4 - PCA of regenotyped and filtered dataset deletions. PCA plots with population (A, C) and dataset (B, D) labels. Upper plots show principal components 5 and 6, while lower plots show principal components 7 and 8.


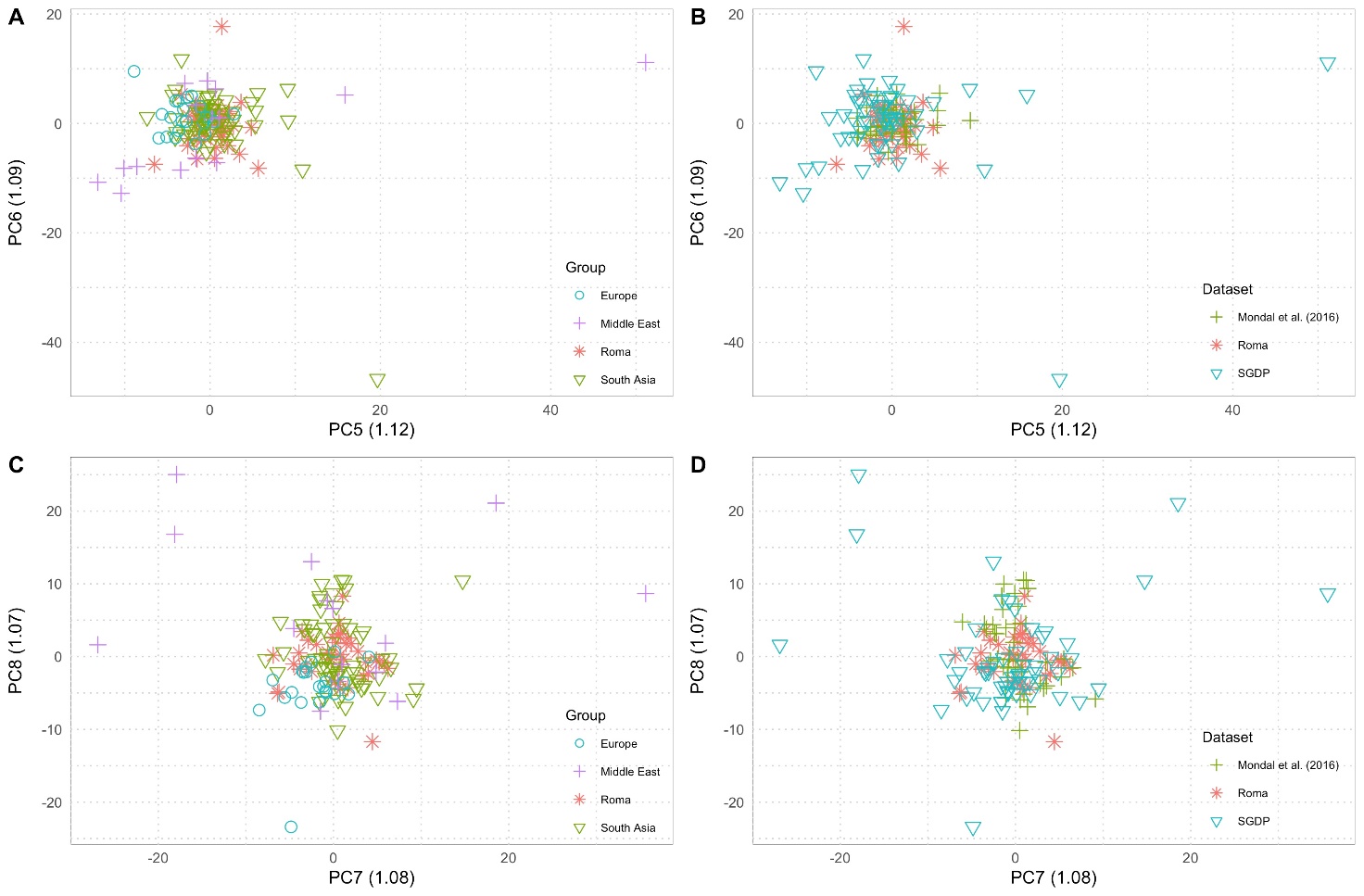


Supplementary Figure 5 - ADMIXTURE analysis of deletions. ADMIXTURE plot using K=2 and K = 3 ancestral populations. Blue, yellow and purple respectively represent West Eurasian, South Asian and Romani ancestries.


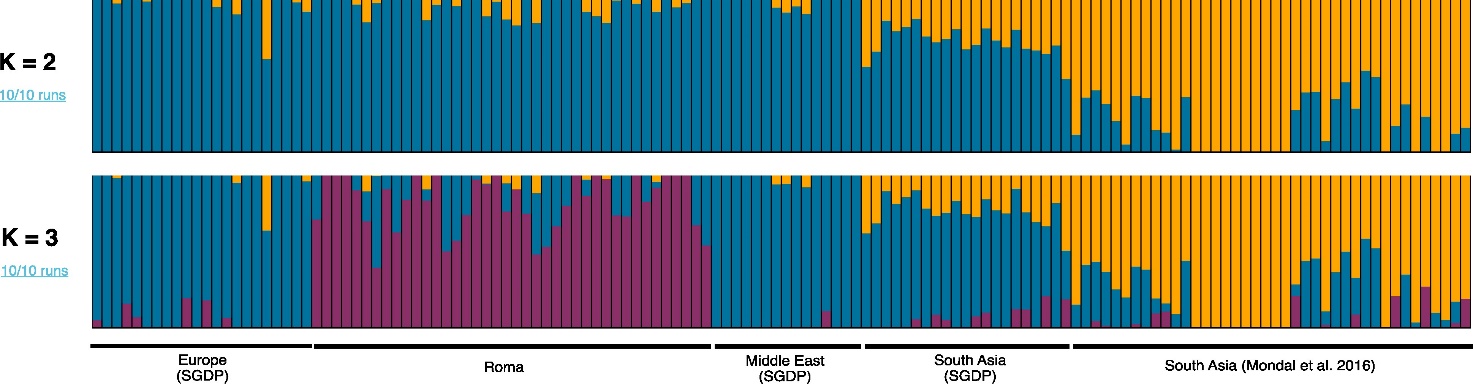


Supplementary Figure 6. PCA obtained with 3171 random SNPs in the same samples of our study, labelled by population (left panel) or source (right panel)


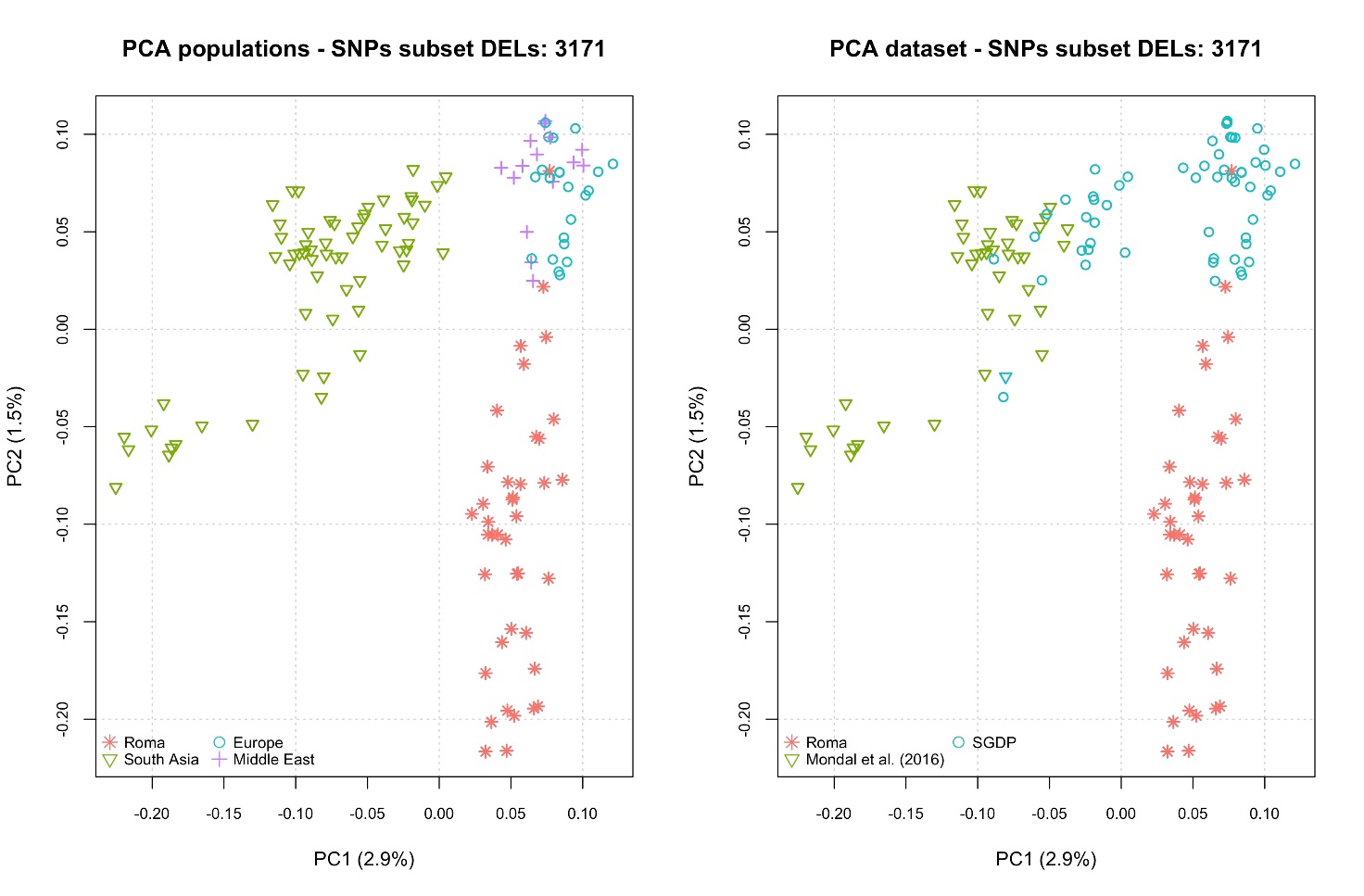


Supplementary Figure 7 - ADMIXTURE analysis of 3171 random SNPs. ADMIXTURE plot using K=2 and K = 3 ancestral populations. Blue, yellow and purple respectively represent West Eurasian, South Asian and Romani ancestries.


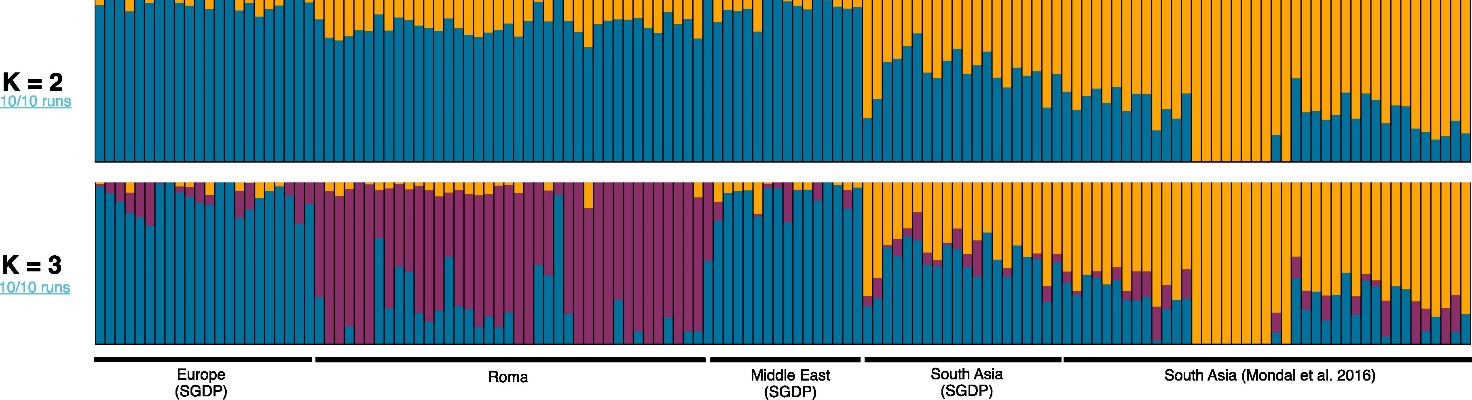


Supplementary Figure 8 - PCA of regenotyped and filtered dataset duplications. PCA plots with population (A, C) and dataset (B, D) labels. Upper plots show principal components 1 and 2, while lower plots show principal components 3 and 4.


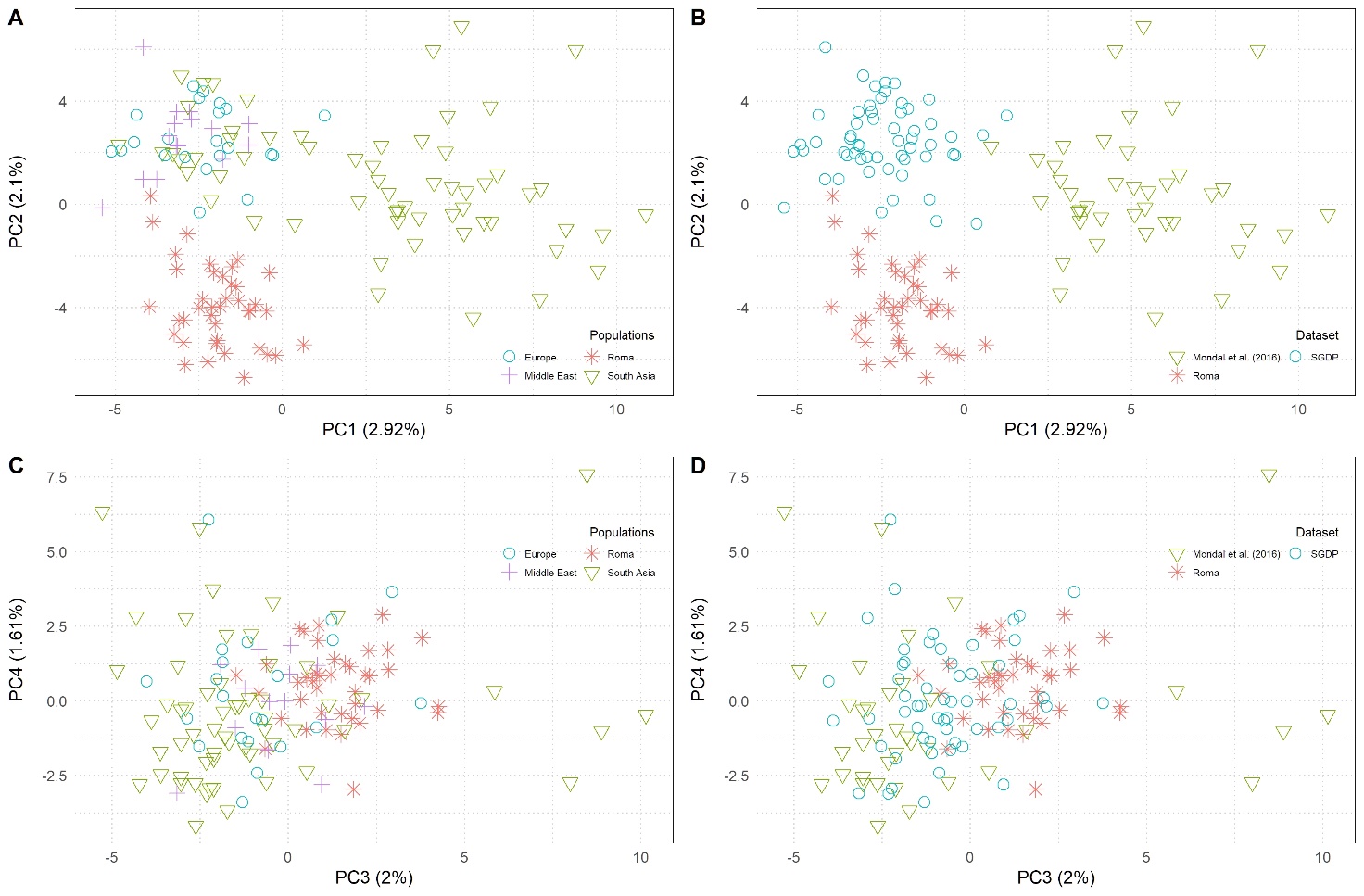


Supplementary Figure 9 - PCA of regenotyped and filtered dataset duplications. PCA plots with population (A, C) and dataset (B, D) labels. Upper plots show principal components 5 and 6, while lower plots show principal components 7 and 8.


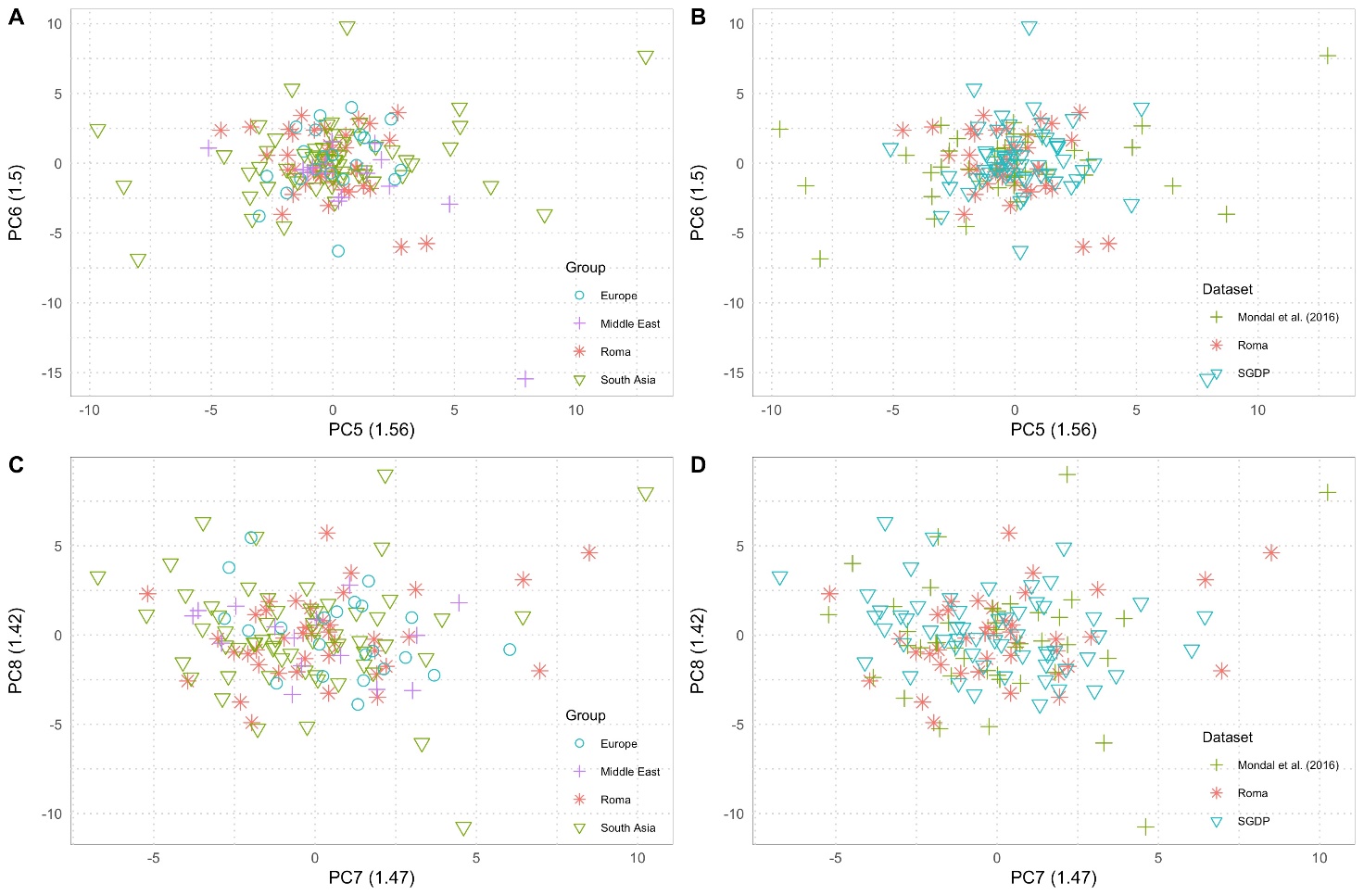


Supplementary Figure 10. Genomic location of the final CNV dataset, by type of CNV (deletions and duplications), and effect (loss-of-function deletions)


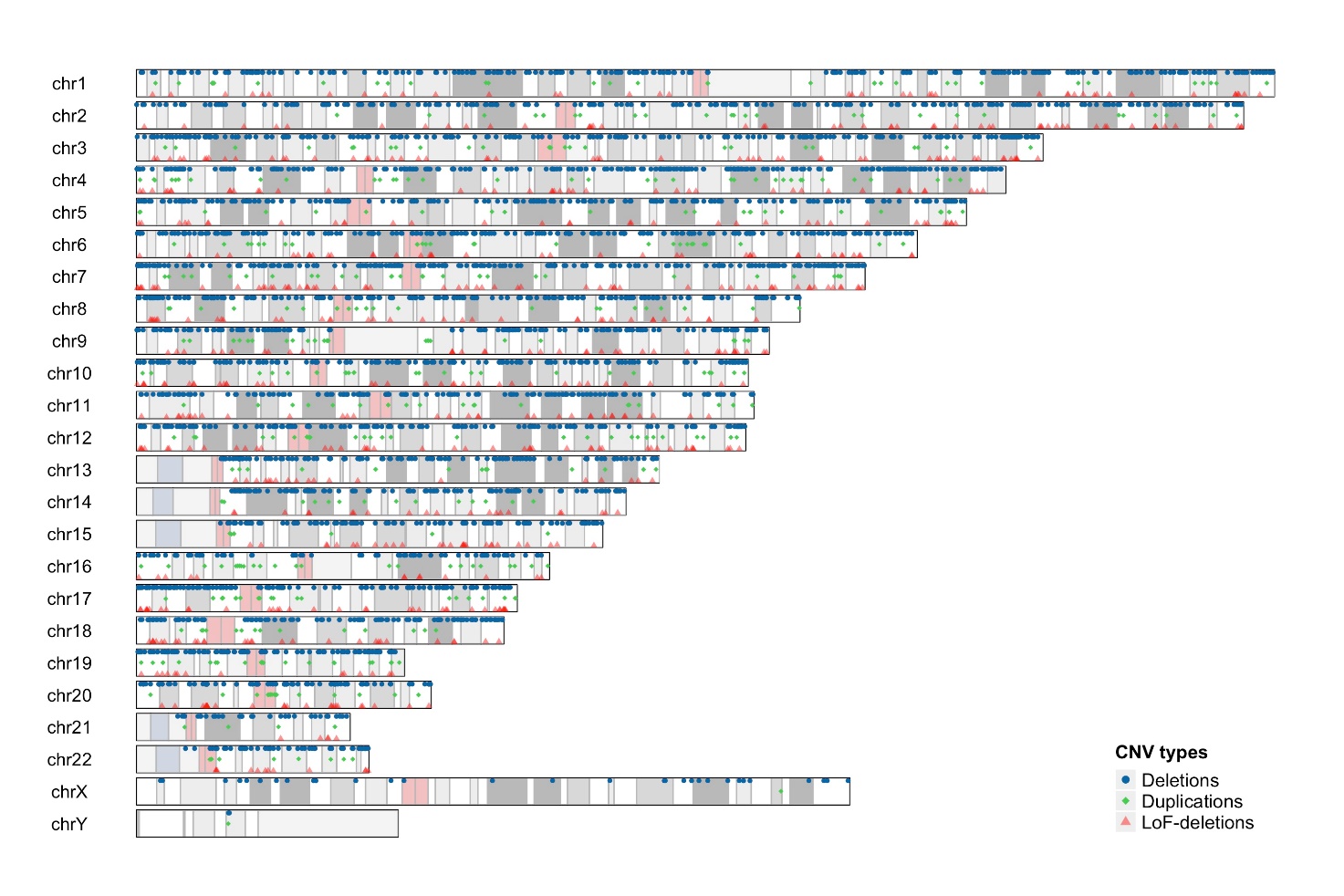


Supplementary Figure 11. Sharing among populations of CNVs, according to their frequency


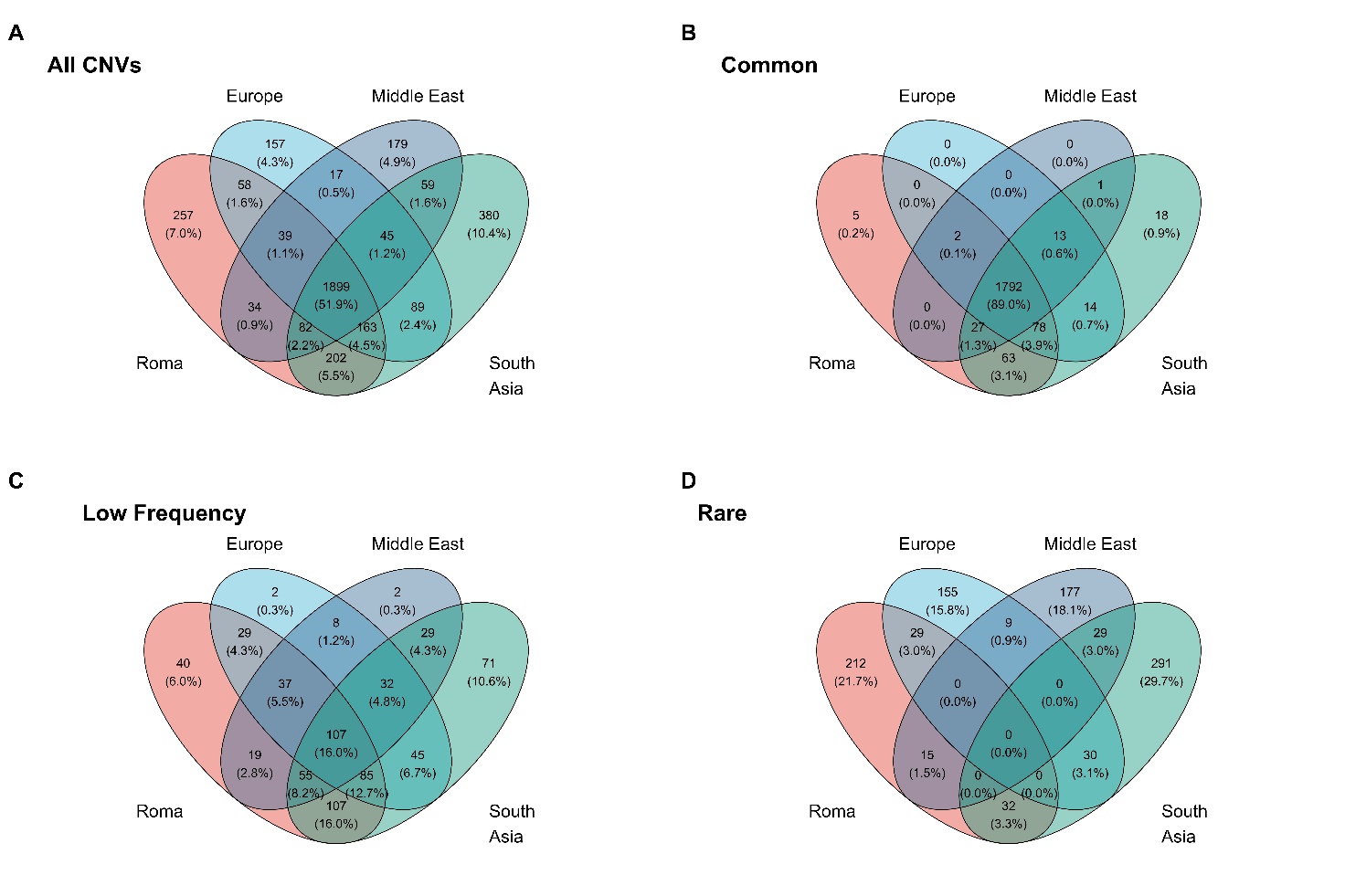


Supplementary Figure 12. Sharing among populations of CNVs according to their genomic location


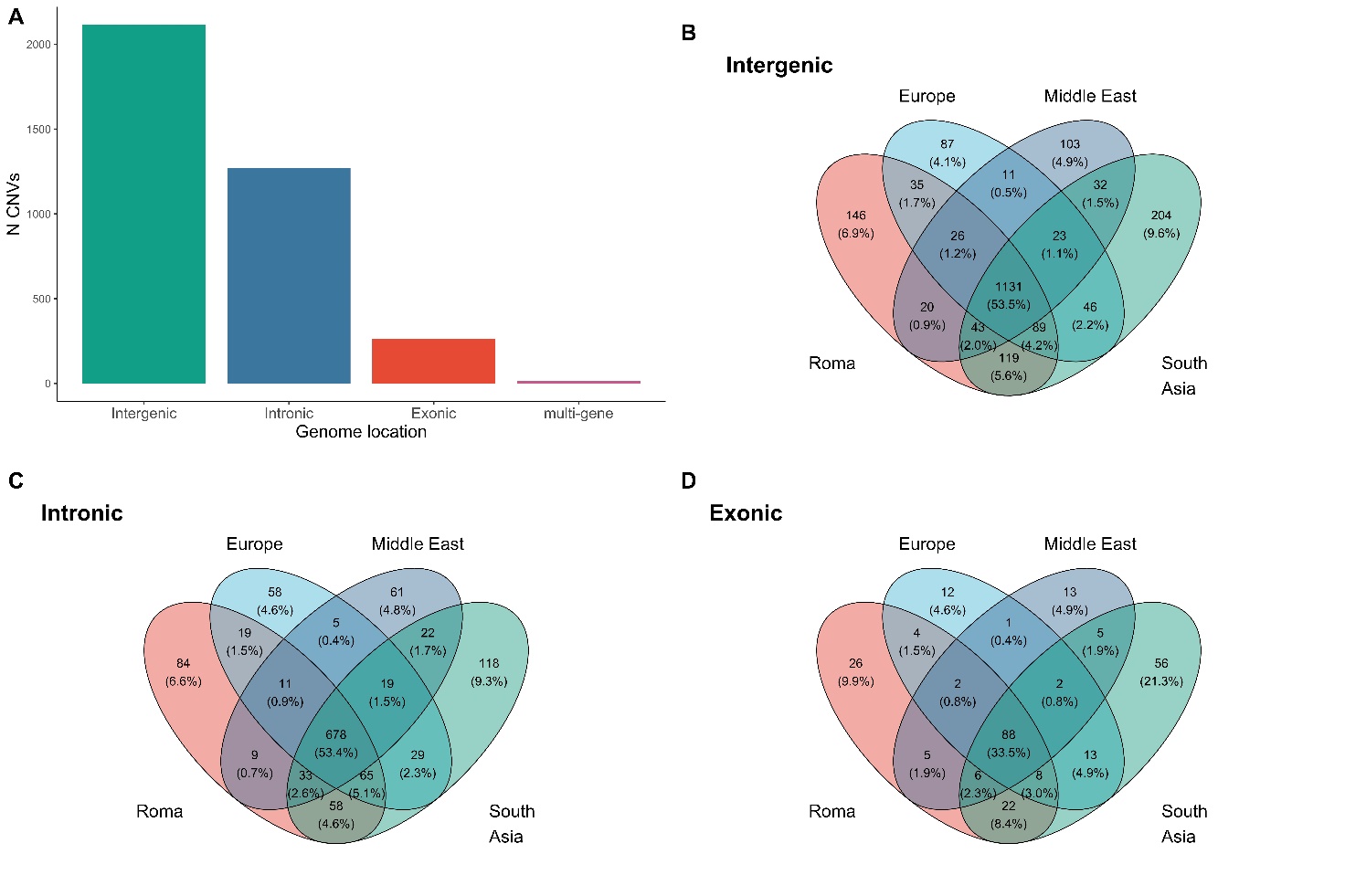


Supplementary Figure 13 - Mean deletion length distributions among populations. Plots show mean deletion length per individual among populations, p-values for ANOVA or Kruskal-Wallis tests and pairwise post-hoc comparisons. The analyses considered all deletions together (A) and intergenic (B), intronic (C) and exonic (D) deletions.


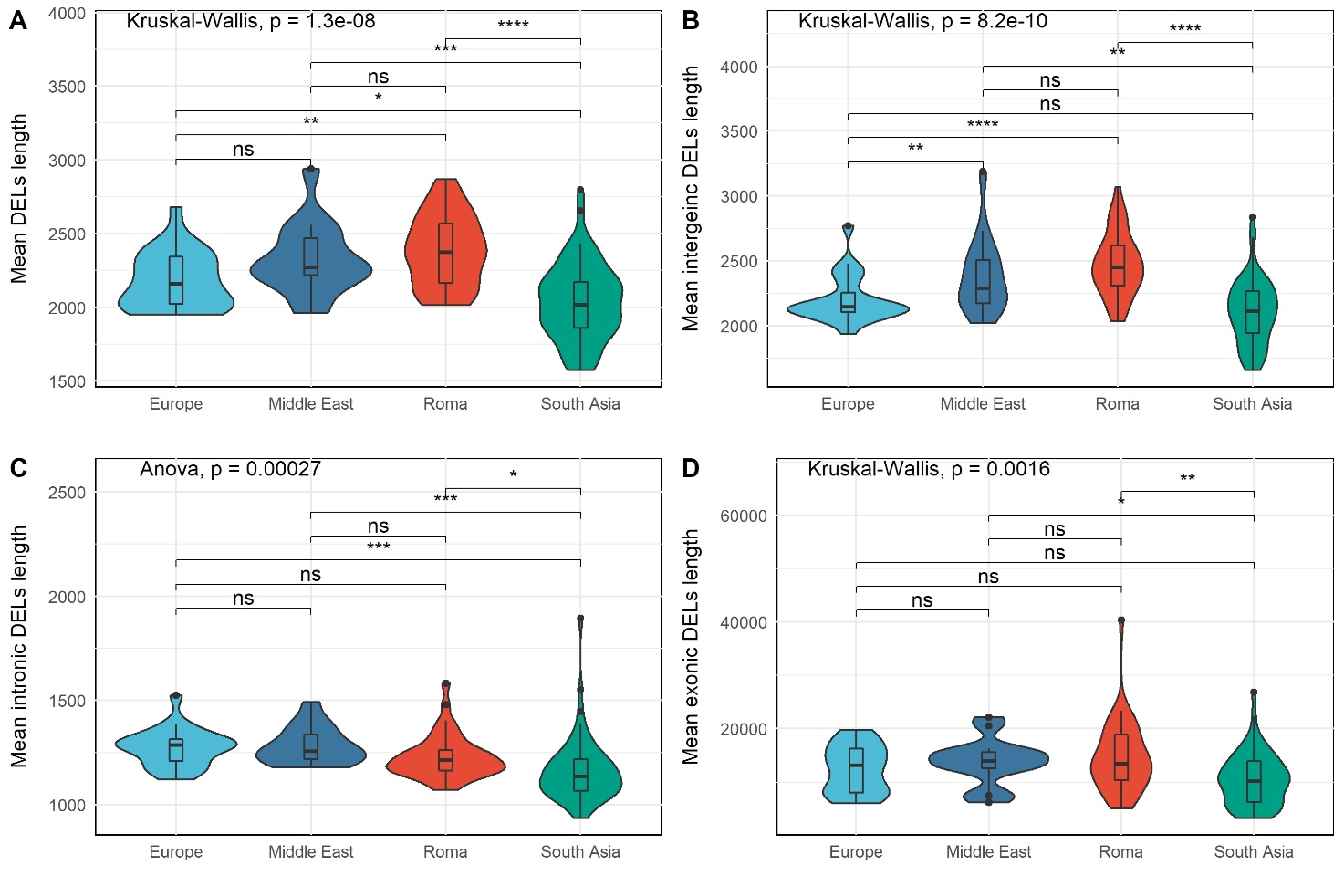


Supplementary Figure 14. Distribution of the number of deletions intersecting OMIM genes


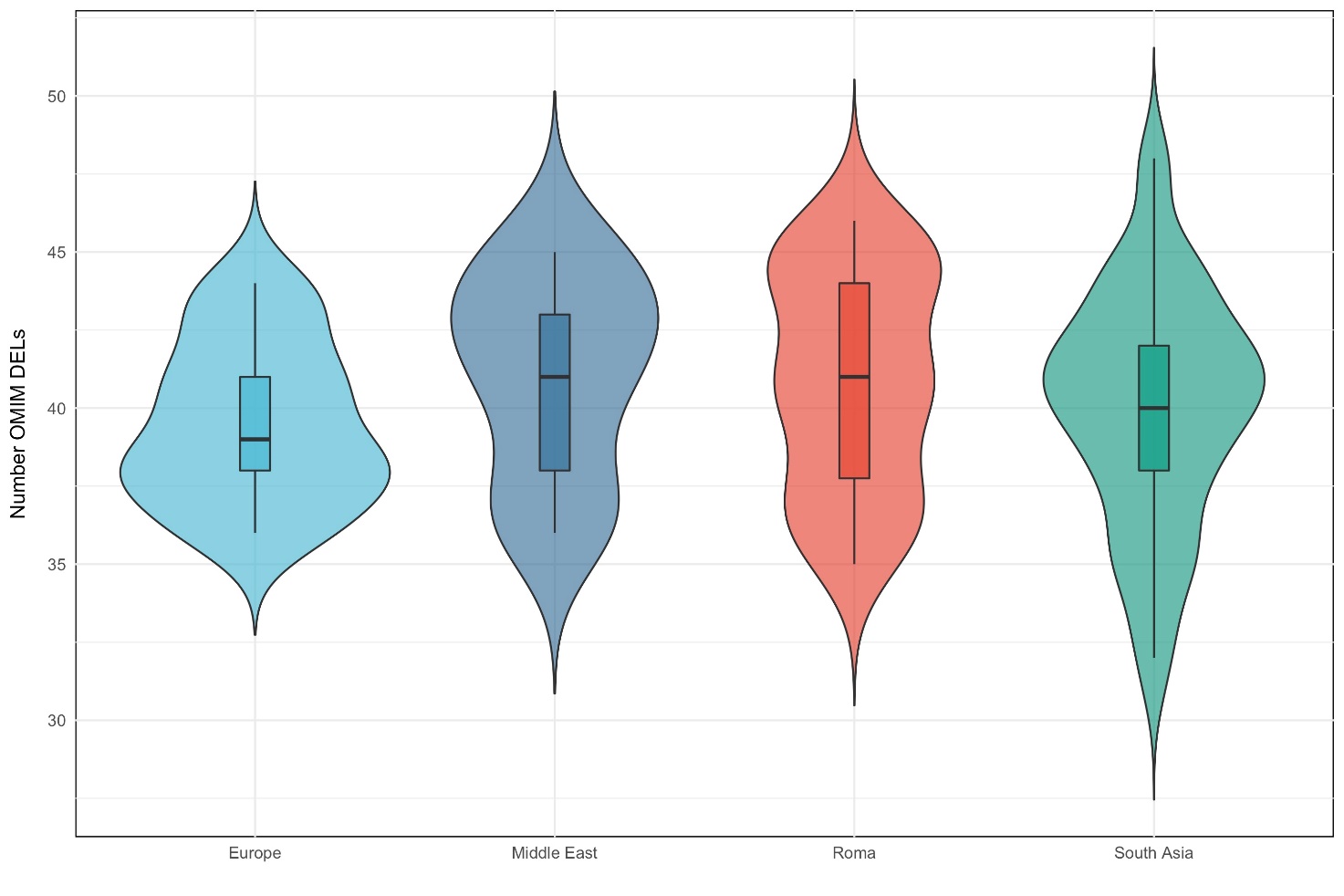


Supplementary Figure 15. Genomic structure of the SHANK (top) and WDPCP (bottom) genes, with their private Roma deletions indicated as red boxes.


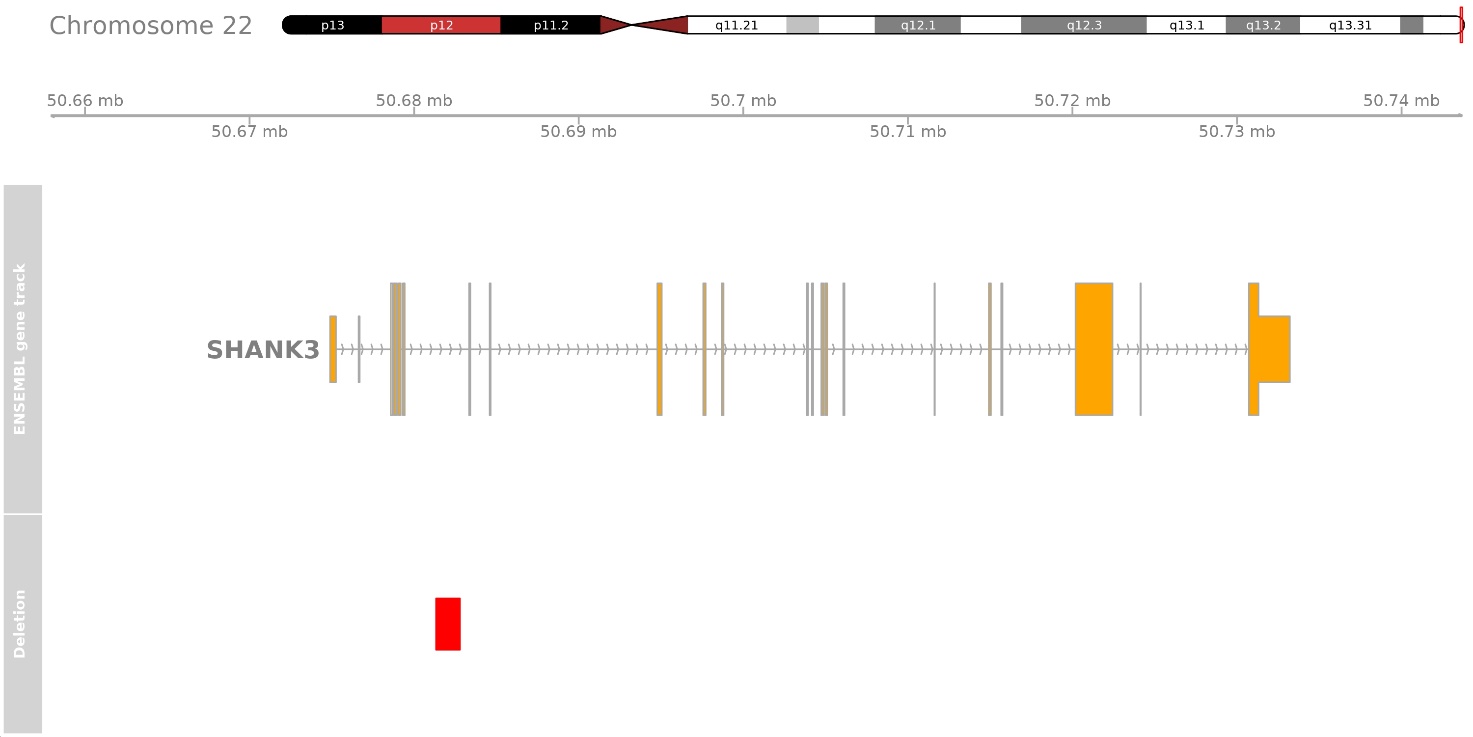


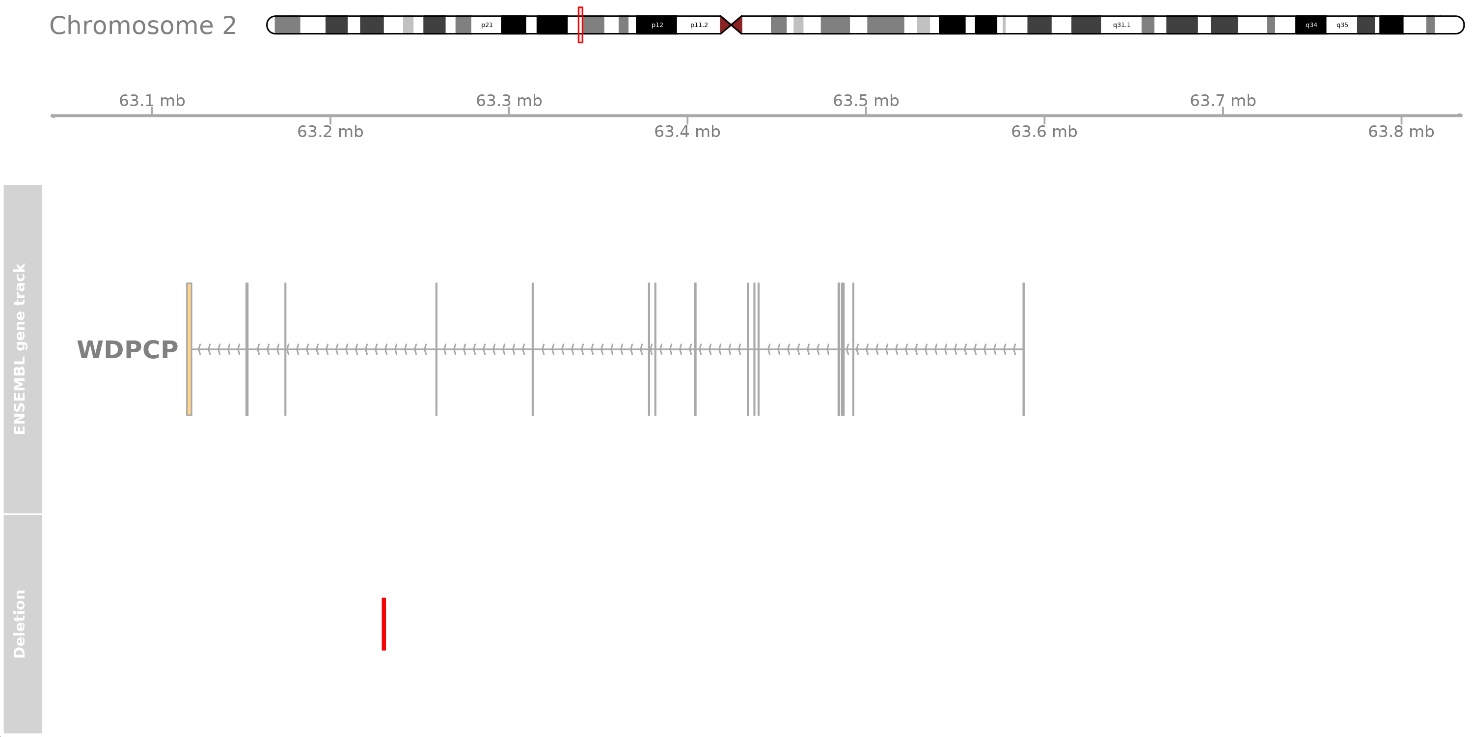

Supplement: Supplementary file 2 — Supplementary file2 (DOCX 3533 KB) [file 439_2023_2579_MOESM2_ESM.docx]
